# The effect of noise on the predictive limit of QSAR models

**DOI:** 10.1186/s13321-021-00571-7

**Published:** 2021-11-25

**Authors:** Scott S. Kolmar, Christopher M. Grulke

**Affiliations:** Center for Computational Toxicology and Exposure, Office of Research and Development, US Environmental Protection Agency, Research Triangle Park, NC USA

**Keywords:** Error, Prediction error, Model evaluation, Gaussian process

## Abstract

A key challenge in the field of Quantitative Structure Activity Relationships (QSAR) is how to effectively treat experimental error in the training and evaluation of computational models. It is often assumed in the field of QSAR that models cannot produce predictions which are more accurate than their training data. Additionally, it is implicitly assumed, by necessity, that data points in test sets or validation sets do not contain error, and that each data point is a population mean. This work proposes the hypothesis that QSAR models *can* make predictions which are more accurate than their training data and that the error-free test set assumption leads to a significant misevaluation of model performance. This work used 8 datasets with six different common QSAR endpoints, because different endpoints should have different amounts of experimental error associated with varying complexity of the measurements. Up to 15 levels of simulated Gaussian distributed random error was added to the datasets, and models were built on the error laden datasets using five different algorithms. The models were trained on the error laden data, evaluated on *error-laden* test sets, and evaluated on *error-free* test sets. The results show that for each level of added error, the RMSE for evaluation on the error free test sets was always better. The results support the hypothesis that, at least under the conditions of Gaussian distributed random error, QSAR models can make predictions which are more accurate than their training data, and that the evaluation of models on error laden test and validation sets may give a flawed measure of model performance. These results have implications for how QSAR models are evaluated, especially for disciplines where experimental error is very large, such as in computational toxicology.

**Supplementary Information:**

The online version contains supplementary material available at 10.1186/s13321-021-00571-7.

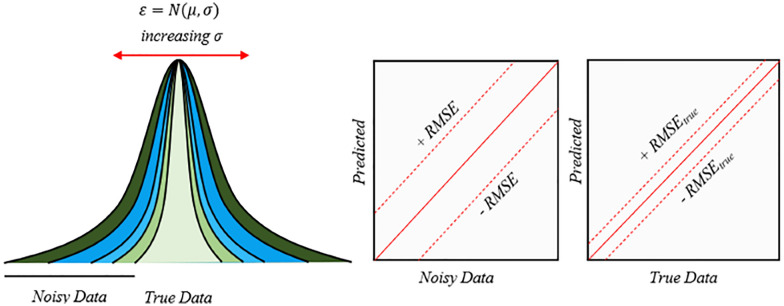

## Introduction

One of the key challenges in Quantitative Structure Activity Relationship (QSAR) modeling is evaluating the predictive performance of models, and evaluation methodology has been the subject of many studies in the past several decades [1–6]. Evaluation of predictive performance has critical implications for the fields of drug discovery [6, 7], toxicological risk assessment [8], and environmental regulation [9], among others. The importance of model evaluation and comparison is reflected in the fourth principle from the Organization for Economic Cooperation and Development (OECD), which states that a QSAR model must have “appropriate measures of goodness of fit, robustness, and predictivity” [9, 10]. While best practice guidelines have often emphasized the need for external validation on compounds that have been rigorously excluded from the training set, implicit assumptions about error in the training and validation data, and how these assumptions might affect performance evaluation, tend to be overlooked [1–3]. It is necessary to examine these assumptions and their effects in order to appropriately evaluate the predictivity of QSAR models and utilize their predictions with confidence.

The most problematic assumption about errors implicitly made during most QSAR modeling is that the given value for any experimental endpoint is the “true” value for that measurement. This assumption is necessarily taken when the following conditions are met: when the endpoint values are represented as single measurements, and when models are compared via their prediction metrics, such as root mean squared error (RMSE) and the coefficient of determination (R^2^). As detailed below, it is often the case that endpoint values are represented as single measurements, and this obligates the modeler to assume that these measurements are representative of the true value. Additionally, the modeler must then compare models using performance metrics that implicitly assume endpoint quantities to be sufficiently representative of physical truth, that is, there is no mathematical mechanism built in to account for the fact that these single values may be several standard deviations away from the actual population mean of that measurement. To put all of this in more rigorous statistical terms, the assumption is made that the given experimental value is the sample mean, and that this sample mean sufficiently approximates the population mean (true value) of all possible measurements [11]. This assumption is made for two main reasons. The first reason is that most models are built on datasets which have only a single, or at best three replicates, for any given measurement and therefore the data does not support a more detailed understanding of the population distribution and uncertainties. For example, an analysis performed on a large set of drug metabolism and pharmacokinetic (DMPK) data showed that 87% of the 358,523 measurements had only a single replicate [7]. Second, most machine learning algorithms, with the exception of Bayesian methods such as Gaussian Process [12] and conformal prediction [13, 14], treat endpoints as discrete quantities rather than distributions thereby forcing QSAR modelers to use only a single value when applying most learning methods. Unfortunately, the assumption that the single experimental value is a good representative of the population mean is often not true. It is unlikely that a measurement’s sample mean will closely approximate the population mean unless the number of replicates is very high [11], although for endpoints which involve fitting a curve to measurements made at multiple concentrations, parametric bootstrapping can provide a workaround to the issue of having few replicates [15]. In sum, the assumption that experimental endpoints are true values ignores the reality that experimental measurements have a distribution and uncertainty associated with them, and this statistical reality has important effects on the predictivity of QSAR models.

**Fig. 1:**
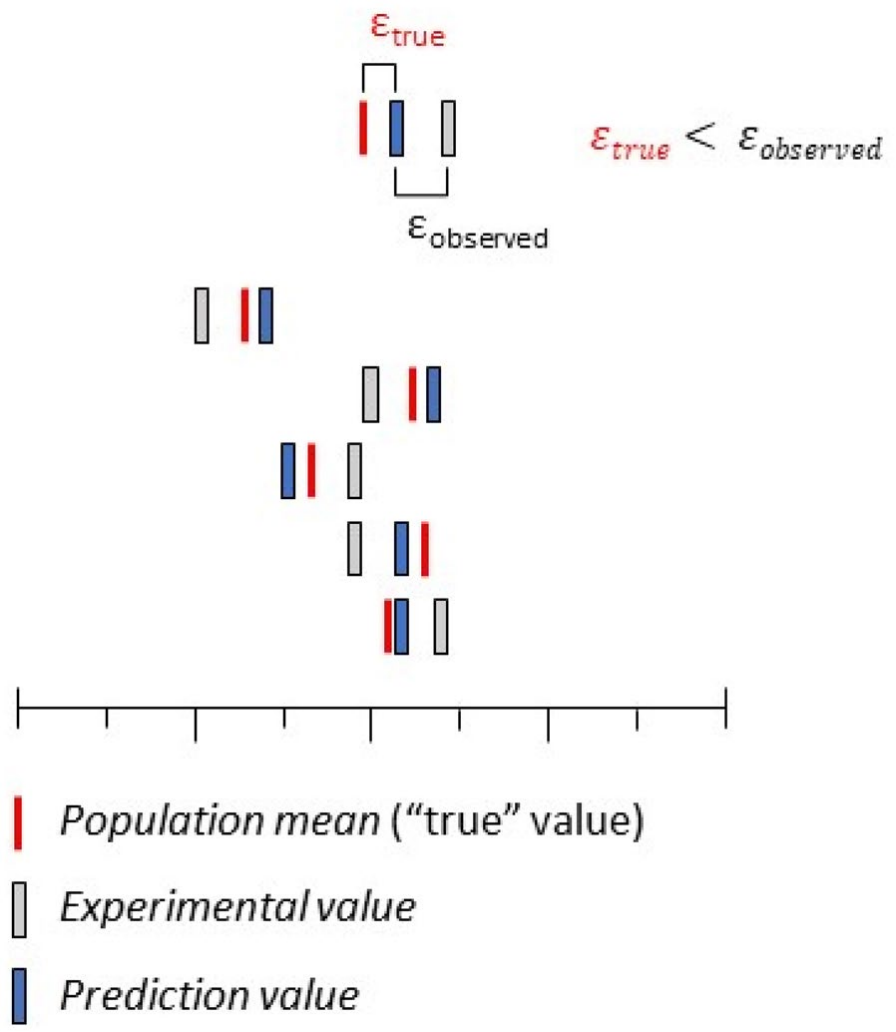
Graphical representation of experimental error and prediction error for an arbitrary dataset. Each row of bars is a separate observation for an arbitrary endpoint. The red bars represent population means (“true” values) for the observation, the grey bars represent experimental values, and the blue bars represent prediction values. If prediction values are closer to the population means than the experimental values, the true error will be smaller than the observed error

Ignoring experimental error of the target property creates two main problems in modeling studies. The first issue is that inaccurate training data may cause a QSAR model to fit the trends in the noise rather than the underlying trends in the data, a well-known phenomenon called overfitting [16]. Overfitting can be diagnosed because performance metrics such as root mean squared error (RMSE) and the coefficient of determination (R^2^) will be far worse for the test set than for the training set [16]. The second and more pernicious issue is that endpoint values in the test set also have experimental error, but these test set values set the standard by which a model is evaluated. If these error laden test set values are used to calculate a model’s performance statistics, then even if a QSAR model predicts close to the true value, the error for that prediction will be observed as high if the experimental test set value is far from the true value (Fig. 1).

Experimental measurements are complicated by two main sources of error. Systematic error biases a measurement in one direction and can be the result of natural or instrumental phenomena [11]. Random error, by definition, is equally likely to affect a measurement in either direction. Systematic error is notoriously difficult, if not impossible, to identify statistically, but random error can be treated effectively using a Gaussian distribution [17]. Experimental error, in the absence of known systematic error, is generally treated to be random. This contention is well supported because variability in natural processes tends to be random, due to contributions from many small underlying factors. The central limit theorem allows us to model random processes using the Gaussian distribution [18]. The sum of this line of reasoning is that most experimental error, in the absence of identifiable systematic error, can be reasonably modeled using a Gaussian distribution. Furthermore, there is a verifiable body of scientific literature (especially in chemistry) which shows that measurements tend to an average value with a Gaussian distribution. [11, 19, 20].

Drawing from this accepted treatment of experimental error, several studies have attempted to better understand the relationship between random experimental error and predictivity. Several works have analyzed proprietary pharmaceutical data and public databases in order to estimate the average error in commonly measured pharmacological and toxicological quantities such as p*K*_*i*_*,* [20] pIC_50_, [19] and cytotoxicity [21]. Brown and coworkers used a computational approach to develop empirical rules for distributions of coefficients of determination (R^2^) based on dataset parameters such as range of endpoint values and number of samples [6]. All three of these studies provide benchmarks to evaluate whether or not the predictivity of any given model is reasonable or not, given what is known about average random error in commonly measured endpoints and how this error propagates to performance statistics such as RMSE and R^2^. A seminal contribution to this topic comes from Cortes-Ciriano and coworkers, in which they performed a full factorial study of random experimental error on 12 different datasets, 12 algorithms, and 10 levels of simulated random experimental error [22]. The results showed that algorithms have different levels of sensitivity to added random experimental error, such that while algorithm A might have a lower RMSE than algorithm B at low noise levels, algorithm A can have a higher RMSE than algorithm B at high noise levels.

A common assertion in the QSAR literature, which is brought up in the studies mentioned above, is that the experimental error in a dataset puts a hard limit on the predictivity of a model, or in other words, that a model cannot make predictions which are more accurate than its training data [7, 20, 23]. The assertion that there is a hard limit on prediction accuracy relies on the assumption that the test set values are true values, but as mentioned above, the test set values also have experimental error. This work poses the main hypothesis that a QSAR model can indeed make predictions which are more accurate than the training data; however, we are unable to validate that these models are better than our test data. This hypothesis is made under the condition that the experimental error is Gaussian distributed; this condition is certainly not representative of every real-world data situation, but it allows the hypothesis to be tested under a set of ideal conditions. A logical method of testing this hypothesis is to compare RMSE_observed_ and RMSE_true_ for a variety of models, which requires model development with two sets of values for each molecule in a dataset, artificially generated error laden experimental values and true values.

Understanding the effect of error on predictivity is particularly important for the field of toxicology, because environmental risk assessments and subsequent regulations depend on the results and confidence intervals of the predictions [24]. Furthermore, toxicological models are often built on in vivo or animal studies measurements, which are notoriously variable due to the myriad factors which contribute to error [8, 25]. It has been posited that variance in the experimental data contributes more to prediction error than the error from the model itself [26, 27]. If the hypothesis that a QSAR model can make predictions which are more accurate than the training data is true, then it would suggest that models trained on highly variable toxicological datasets could produce accurate and therefore reliable predictions. This work will test this hypothesis and discuss the results in the context of toxicological datasets.

## Methods

### Experimental design

Residual error in a model prediction for a validation compound can be understood in two different ways. Based on the assumption that the experimentally measured values are true, the error is calculated as simply the difference between the observed value and the predicted value, ε_observed_. However, the error of interest for a predictive model is actually the difference between the population mean and the predictive value, ε_true_ (Fig. 1). While this argument conforms with our understood goals for a QSAR model, population means are difficult to ascertain for most endpoints of biological relevance, and therefore, ε_true_ is often out of reach. However, if a computational experiment made it possible to determine ε_true_, it would allow us to investigate the question of whether there is a hard limit on the predictivity of a model, or if the limit is actually on our ability to accurately measure the predictivity of the model.

If *Y* is the vector of experimental endpoints, *Z* the vector of true values, and *Ŷ* the vector of model predictions, then *ε*_*i*_ is the difference between an experimental measurement and the true value, *RMSE*_*observed*_ is the prediction error calculated from the experimental endpoints, and *RMSE*_*true*_ is the prediction error calculated from the true values. *RMSE*_*observed*_ will be higher than *RMSE*_*true*_ if the average *ε* is large. The problematic assumption is that *ε* is assumed to be 0. This means that *RMSE*_*observed*_ is often mistaken for *RMSE*_*true*_ when evaluating a QSAR model, and thus the true predictivity of a model is probably underestimated.

1 $$Y = \left( {y_{1} , \ldots ,{ }y_{k} } \right)$$

2 $$Z = \left( {z_{1} , \ldots ,{ }z_{k} } \right)$$

3 $${\hat{\text{Y}}} = \left( {{\hat{\text{y}}}_{1} , \ldots , {\hat{\text{y}}}_{k} } \right)$$

4 $$\varepsilon_{i} = \left( {y_{i} - z_{i} } \right)$$

5 $$RMSE_{observed} = \sqrt {\frac{{\mathop \sum \nolimits_{i = 1}^{k} ({\hat{\text{y}}}_{i} - y_{i} )^{2} }}{k}}$$

6 $$RMSE_{true} = \sqrt {\frac{{\mathop \sum \nolimits_{i = 1}^{k} ({\hat{\text{y}}}_{i} - { }z_{i} )^{2} }}{k}}$$

As mentioned above though, evaluating this claim requires having both true values and experimental measurements to assess whether RMSE_observed_ is greater than RMSE_true_. In the absence of such true values, we assume that for the datasets used in this experiment, the original values are the true values and then create simulated “experimental” observations adding increasing amounts of Gaussian error to those “true” values. This assumption (which directly contradicts our premise that doing so is dangerous) and its possible ramifications will be addressed in the “Data sets” and “Discussion and conclusion” sections.

While the effect of error (including the addition of simulated error) has been studied in previous publications by Cortes-Ciriano and co-workers [22], this work has some key differences which are important to highlight. The purpose of the previous work was to systematically explore how different algorithms respond to simulated error in order to benchmark performance. The authors achieved this by modeling 12 different protein pIC_50_ datasets with 12 algorithms, and by observing the increase in RMSE as simulated Gaussian distributed error is added to these datasets. While the datasets showed a diversity of targets, the type of endpoint, pIC_50_, is the same for each dataset. Additionally, the range of estimated native experimental error for these datasets is only 1.1 log units. In contrast, the objective of the present work is to test the hypothesis that a QSAR model can predict more accurately than the dataset on which it is trained. The present approach, similarly, is to use several common algorithms to model different datasets and observe how the addition of simulated Gaussian distributed error affects the RMSE. A key difference here, however, is to compare the performance statistics for a model’s prediction on the noisy data vs a model’s prediction on the true data in an effort to de-couple the potential causes of observed prediction error and assess their individual impacts on our observed model performance. The hope is to be able to separate and better understand three potential causes of error: learning error (i.e., prediction error caused by the modeling methodology being insufficient), propagated training set error (i.e., prediction error caused by the training set having errors that are then learned by the model), and validation error (i.e., prediction “error” that is perceived due to the validation set itself having error). Here, *RMSE*_*0*_ can be understood as learning error; *RMSE*_*true*_*—RMSE*_*0*_ would approximate propagated training error; and *RMSE—RMSE*_*true*_ would approximate the validation error.

### Data sets

All data sets have error associated with their target properties, including random experimental error and systematic error. However, the magnitude of random experimental error and the nature of systematic error varies widely with the type of endpoint. For this study, we primarily consider the differences in error characteristics likely within five categories of endpoints: quantum mechanical calculations, physicochemical properties, biochemical binding, in vitro bioactivity, and in vivo toxicity. While quantum mechanical calculations do not have random experimental error, because the same calculation will give exactly the same number [28], systematic error is prevalent and comes from the fundamental choice of exchange–correlation approximations used in the density functional theory (DFT) method [29]. Measurements of physiochemical properties typically require determination of equilibrium concentrations of compounds in various solvents or phases [30], and are often made with standard analytical chemistry methods such as liquid chromatography, gas chromatography, or spectroscopy [31]. The random experimental and systematic error associated with these measurements thus comes from factors such as the purity of the compounds, instrument calibration, and instrument sensitivity [11]. In contrast, factors in biochemical measurements, such as protein purity, accurate determination of protein concentration, and equilibration time contribute to higher random experimental and systematic error that can make these measurements highly variable [32]. Toxicological datasets can include many different types of in vitro bioactivity and in vivo measurements, which are sometimes aggregated in order to provide composite scores for use in classification problems [33, 34]. These datasets likely have the highest level of random experimental and systematic error because the sources of error are diverse and the accumulated errors propagate. Utilizing datasets from each of these categories allows a comparison to be made between datasets with which are likely to have increasing amounts of native random experimental error thereby allowing us to investigate how our assumption regarding the “truth” of the values provided in the dataset affects our conclusions.

The majority of quantum mechanical [35, 36], physiochemical [38], and biochemical data [39] sets included in this analysis were taken from MoleculeNet [40], a large curated collection of chemical data which is intended to provide standard benchmarking data sets for QSAR models. As the primary goal of this work is to benchmark different common QSAR algorithms, the MoleculeNet collection provides several high-quality data sets for comparison. In vitro bioactivity sets were obtained from the EPA’s ToxCast [34] database and in vivo toxicity datasets were represented by an LD_50_ data set gathered a report by Gadeleta and coworkers; 75% of these LD_50_ values were taken from the EPA’s DSSTox database, with the other 25% assembled from literature publications as described in Gadeleta et al. [41] A summarization of the datasets used is available in Table 1. Additional dataset details are described in Additional file 1.

**Table 1:** Datasets used in this work, with the number of molecules, endpoint, endpoint units, range, and reference for each

Dataset	Category	Entries^a^	Endpoint	Range	Refs.
G298_atom	Quantum mechanical	131,082	ΔG^o^_at_ (kcal mol^−1^)	− 2417to − 288	[29, 30]
Alpha	Quantum mechanical	131,082	α (Bohr^3^)	9.0 to 27.8	[29, 30]
Lip	Physiochemical	4200	logD	− 1.5 to 4.5	[31]
Solv	Physiochemical	642	ΔG^o^_hyd_ (kcal mol^−1^)	− 25.5 to 3.4	[32]
BACE	Biochemical	1513	pIC_50_	2.5 to 10.5	[33]
Tox_102^b^	Toxicological in vitro	971	logAC_50_	− 2.1 to 4.7	[28]
Tox_134^c^	Toxicological in vitro	1347	logAC_50_	− 4.0 to 2.8	[28]
LD_50_	Toxicological in vivo	5003	logLD_50_ (mg kg^−1^)	− 1.9 to 4.8	[35]

^a^Original size of the dataset. If datasets have more than 1000 molecules, they were randomly sampled down to a size of 1000 before modeling

^b^Includes data exclusively from the ATG-PPre-cis assay

^c^Includes data exclusively from the ATG-PPARg-trans assay

### Descriptors

Molecular descriptors were generated using PadelPy [42], a python package which wraps the Padel descriptor software [43, 44], or with OPERA, an open source software package which also generates Padel descriptors [45, 46]. The Padel software generates up to 1,875 descriptors, including 1444 1D/2D, and 431 3D descriptors. These quantities include electrotopological, topochemical, and linear free energy descriptors, as well as ring counts, McGowan volume, Crippen’s logP, and others. While there are many choices of descriptor sets [4, 47, 48], Padel descriptors are commonly used in QSAR workflows and thus provide a logical and reasonable method for performing a proof of concept study such as the work presented here. For this work, only the 1,444 1D and 2D Padel descriptors were used.

### Modeling workflow

**Fig. 2:**
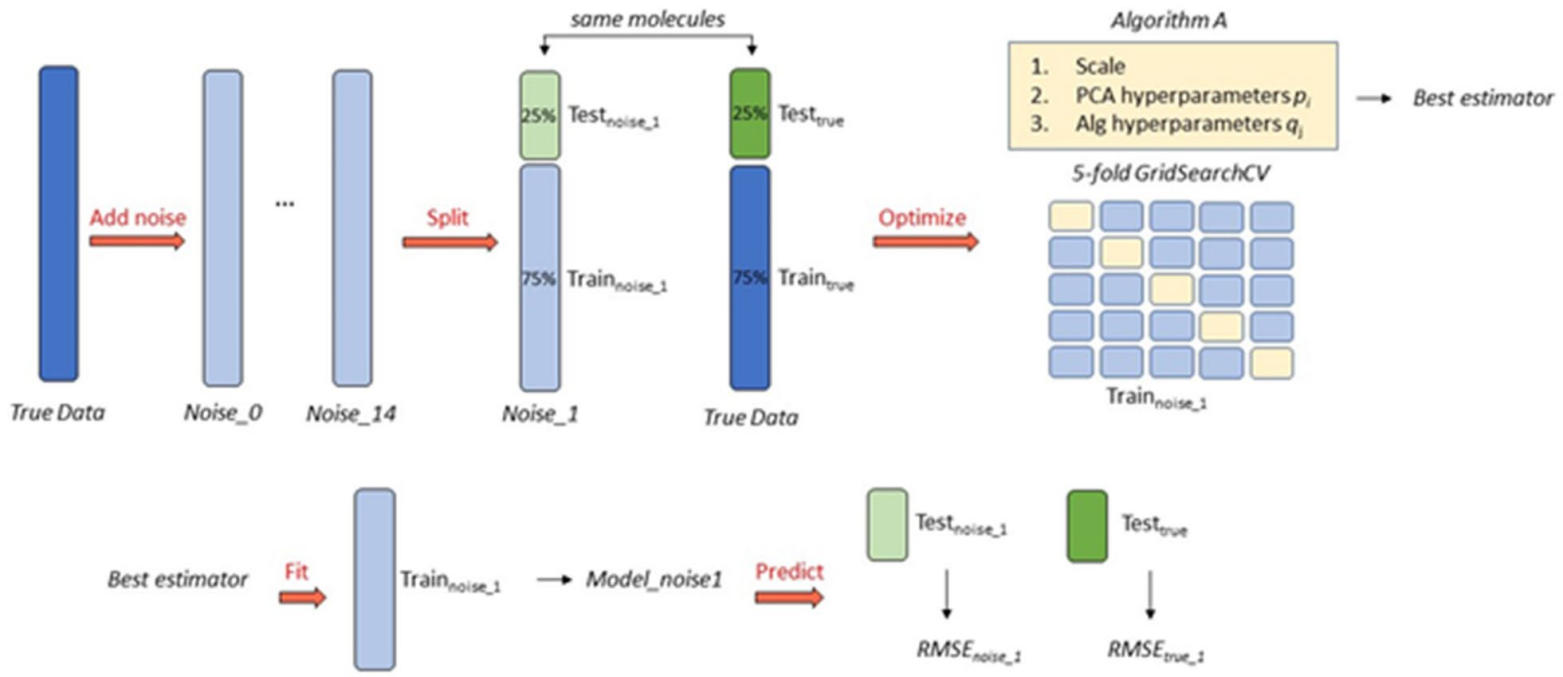
The modeling workflow and machine learning pipeline which is used in this work. Fifteen noisy datasets are first generated by adding Gaussian distributed noise to the dataset. Each of these noisy datasets is then split into Test_noise_n_ and Train_noise_n_, and the true data is split into Test_true_ and Train_true_ (no training is performed on Train_true_). The hyperparameters for PCA and the chosen algorithm are then optimized using GridSearchCV (or RandomSearchCV) to give a *Best estimator*. The *Best estimator* is then fit on Train_noise_n_ to give *Model_noise*_*n*_, and *Model_noise*_*n*_ predicts on both Test_noise_n_ and Test_true_ to give RMSE_noise_n_ and RMSE_true_n_. This procedure is repeated for each noisy dataset

Padel descriptors were first generated using PadelPy or OPERA using a SMILES string for each molecule [49]. In some cases, a subset of descriptors (up to 12) had infinite values, in which case those descriptors were removed from the dataset. If the dataset has more than 1000 molecules, it was sampled down to a size of 1,000; if the dataset has less than 1000 molecules, it continued without sampling. Custom code was written in python, utilizing the popular machine learning package scikit-learn [50], to run the dataset through a machine learning pipeline. The code implemented the following workflow on each dataset. The endpoint data column was used to generate 15 additional endpoint data columns with increasing levels of gaussian distributed noise. This process was repeated five times at each noise level to give 75 total datasets. A machine learning algorithm was chosen, such as k-nearest neighbors (kNN) or random forest (RF). The algorithm was then preprocessed, optimized, trained, and fit on each of the 75 datasets with added noise, giving 75 unique models, 75 RMSE’s, and 75 R^2^’s. Each of these models was then fit on the original dataset which has no added noise, giving an additional 75 RMSE_true_’s and 75 R^2^_true_’s. The RMSE, RMSE_true_, R^2^, and R^2^_true_ values were then plotted against noise level. This process was repeated for each algorithm and each dataset. Details on each step of this process are given below. A graphical representation of the modeling workflow and machine learning pipeline is provided in Fig. 2.

### Machine learning pipeline

Prior to modeling a given dataset, 25% of the data was split into a test set, and 75% of the data was split into a training set. Each algorithm was then put through a pipeline of steps before training on the training set and predicting on the test set. This pipeline consisted of three steps: scaling, principal component analysis (PCA), and algorithm fitting, with PCA and algorithm hyperparameters optimized using fivefold GridSearchCV or RandomSearchCV. Scaling was applied to all features (descriptor values) using StandardScaler, which centers each feature on the mean and scales to unit variance, which is a common requirement for many algorithms. Dimension reduction was then applied using PCA, optimizing the number of principal components. Algorithm hyperparameters were then optimized as shown in Table 2.

**Table 2:** Algorithms used in this work and their respective hyperparameter optimization spaces

Algorithm	Hyperparameters searched in optimization^a,b^
Ridge regression (Ridge)	*PCA n components* $\in \left( {1,{ }3,{ } \ldots { },59} \right)$ α $\in \left( {1, 2, 3, 4, 5, 10} \right)$
k-nearest neighbors (kNN)	*PCA n components* $\in \left( {1,{ }3,{ } \ldots { },59} \right)$ *k* $\in \left( {1,{ }2,{ } \ldots ,{ }20} \right)$
Support vector regressor (SVR)	*PCA n components* $\in \left( {1,{ }3,{ } \ldots { },59} \right)$ *C* $\in \left( {0.01,{ }0.1,{ }1,{ }10} \right)$ *kernel:* radial basis function (RBF)
Random forest (RF)	*PCA n components* $\in \left( {1,{ }3,{ } \ldots { },59} \right)$ *n estimators* $\in \left( {1,{ }10,{ } \ldots ,{ }200} \right)$ *max depth* $\in \left( {1,{ }3,{ } \ldots ,{ }99} \right)$ *max leaf nodes* $\in \left( {2,{ }12,{ } \ldots ,{ }92} \right)$
Gaussian process (GP)	*PCA n components* $\in \left( {1,{ }3,{ } \ldots { },59} \right)$ *kernel*:^c^ RBF, WhiteKernel, Matern, DotProduct, ExpSineSquared, ConstantKernel or RationalQuadratic *Normalize y*: true

^a^Ridge, kNN, SVR, and GP algorithms were optimized using fivefold *GridSearchCV*, but RF was optimized using fivefold *RandomSearchCV* with 500 iterations

^b^All algorithm hyperparameters which are not listed in this table were set to the defaults provided in the sci-kit learn library

^c^For most datasets, only a single kernel converged. So the kernel was not optimized in GridSearchCV, it was chosen beforehand and used for the entire dataset

### Random error generation

Random error was added to datasets by sampling from a Gaussian distribution of zero mean and increasing standard deviation σ_noise_. Noise was added only to the target variables and not to the descriptors. This σ_noise_ was determined from the product of the range of endpoint values in the dataset, the noise level *n,* and a multiplier. This multiplier was set to 0.01 after experimentation with a range of values and observing the effect on RMSE. Each dataset was used to generate 15 noise levels with 5 replicates at each noise level. Because *n* starts at 0, the 0th noise level has no added noise.

7 $$Y_{{noise_{n} ,i}} { } = Y + N\left( {0,{ }\sigma_{{noise_{n} }} } \right)$$

8 $$\sigma_{{noise_{n} }} = \left( {Y_{max} - Y_{min} } \right){*}multiplier{*}n$$

$$n{ } \in \left( {0, \ldots ,{ }14} \right)$$

$$i{ } \in \left( {1, \ldots ,{ }5} \right)$$

#### Algorithms

Several machine learning algorithms were chosen for this study which are common to QSAR modeling workflows and which represent a variety of mathematical approaches for capturing complex patterns in data. Applying this analysis to a selection of algorithms allows us to determine whether the ability to make predictions which are more accurate than the training data is conserved across a variety of methods.

#### Ridge regression

Ridge regression is a form of linear regression that utilizes a technique called regularization in order to reduce model complexity and minimize overfitting [51]. If a dataset has *n* number of features *x*, then a linear model calculates a prediction *ŷ* as a function of *n* number of weight coefficients *β* times *x* (Eq. 9)*.* The resulting cost function for this linear model simply minimizes the squared difference between predictions *ŷ* and observations *y* by adjusting *β* (Eq. 10). Ridge regression adds a regularization term to this cost function which contains a regularization coefficient *λ* times the square of each weight coefficient *β* (Eq. 11). This *λ* is set as a hyperparameter for the ridge regression algorithm. The larger *λ* is, the more a particular coefficient will be dampened by the cost function. This means that if some feature *x*_*i*_ is dominating the linear model, causing overfitting, the weight coefficient will be dampened and the influence on the model will be reduced.

9 $${\hat{\text{y}}} = \beta_{0} + \beta_{1} x_{1} + \ldots + \beta_{n} x_{n}$$

10 $$L = \mathop \sum \limits_{i = 1}^{M} \left( {{\hat{\text{y}}} - {\text{y}}} \right)^{2} = \mathop \sum \limits_{i = 1}^{M} \left( {{\hat{\text{y}}} - \mathop \sum \limits_{j = 0}^{n} \beta_{j} x_{ij} } \right)^{2}$$

11 $$L = \mathop \sum \limits_{i = 1}^{M} \left( {{\hat{\text{y}}} - {\text{y}}} \right)^{2} = \mathop \sum \limits_{i = 1}^{M} \left( {{\hat{\text{y}}} - \mathop \sum \limits_{j = 0}^{M} \beta_{j} x_{ij} } \right)^{2} + \lambda \mathop \sum \limits_{j = 0}^{n} \beta_{j}^{2}$$

#### K nearest neighbors

KNN regression [52] uses distance measures to find the *k* observations which are closest to the coordinates of the input features in *n* dimensional vector space. The average observation value of these *k* neighbors is used to calculate the prediction *ŷ*. Each observation *y*_*i*_ of the *k* nearest neighbors can also be weighted by the distance *D*_*i*_ (Eq. 12)*.* The most common distance measure to use is Euclidean distance (Eq. 13), which was used in this work.

12 $${\hat{\text{y}}} = \frac{1}{k}\mathop \sum \limits_{i = 1}^{k} D_{i} y_{i}$$

13 $$D = \sqrt {\mathop \sum \limits_{i = 0}^{n} \left( {p_{i} - q_{i} } \right)^{2} }$$

#### Support vector machines

Support vector machine (SVM) methods [53] are non-parametric algorithms which rely instead on kernel functions to make predictions. SVM’s predict complex non-linear trends by transforming the *n* dimensional input vector space into a higher *m* dimensional vector space. This is achieved by a mapping function, otherwise known as a kernel function *k(****x****, ****x’****)* which acts on the vectors ***x*** and ***x’***. Once the input vectors are in the higher dimensional space, a linear hyperplane can be drawn to separate the data by maximizing the margin between each data point and the hyperplane. This hyperplane is a function of the input vector ***x*** and the weight vector ***β*** (Eq. 14). The linear form of this hyperplane can be learned by minimizing the cost function J (Eq. 15). When a kernel function is applied to transform ***x*** into a higher dimensional space, and when we define the weighting coefficient vector ***β*** by a linear combination of the training observations (Eq. 16), we arrive at the new functional form for support vector regressor (SVR) (Eq. 17).

14 $${\hat{\text{y}}} = \beta_{0} + \beta_{1} x_{1} + \ldots + \beta_{n} x_{n} = {\varvec{\beta}} \cdot {\varvec{x}}$$

15 $$J\left( {\varvec{\beta}} \right) = \frac{1}{2}{\varvec{\beta}}^{{\varvec{T}}} {\varvec{\beta}}$$

16 $$\beta = \mathop \sum \limits_{i = 0}^{n} a_{i} x_{i}$$

17 $${\hat{\text{y}}} = \mathop \sum \limits_{i = 0}^{n} a_{i} y_{i} K\left( {{\varvec{x}}_{{\varvec{i}}} , {\varvec{x}} } \right)$$

#### Random forest

The Random Forest (RF) algorithm [54] is an ensemble method which makes predictions from the average of many individual decision trees predictions. The RF algorithm uses bagging with replacement to create *n* samples from a dataset and builds a decision tree on each of those bagged samples, creating a “forest” of random decision trees. The features, or input variables *x,* can also be sampled during this process. This approach reduces the common problem of overfitting with decision trees. In Eq. 18, *T*_*i*_*(****x****)* is an individual decision tree trained on a subset of the input variable vector **x**, and there are B decision trees.

18 $${\hat{\text{y}}} = \frac{1}{B}\mathop \sum \limits_{i = 0}^{B} T_{i} \left( {\varvec{x}} \right)$$

## Results

### RMSE response to error

Each dataset was used to generate 15 levels of noise with five replicates at each noise level, and the ridge, kNN, SVR, and RF algorithms were used to model each dataset with the various levels of added noise. These noisy data simulate the real-world situation in which the experimental data has large amounts of random experimental error. Algorithms are optimized and trained on Train_noise_, then the resulting model predicts both Test_noise_ and Test_true_ sets. The quantities RMSE_noise_ and R^2^_noise_ are the metrics of the predicted values versus Test_noise_ data, replicating the real-world situation where test/validation sets have the same level of noise as the training data. The quantities RMSE_true_ and R^2^_true_ are the metrics of the predicted values versus Test_true_ data, our presumed “true” endpoint values. Therefore, the RMSE_noise_ reports on the ability of the algorithm, which is trained on Train_noise_, to predict the noisy values in Test_nois*e*_. In contrast, the RMSE_true_ reports on the ability of the algorithm to predict the values in Test_true_, which have no added noise and thus represent what we can define as “true” values. In this experimental design, for a given noise level, if RMSE_true_ is lower than RMSE_noise_, then the model has made *fewer* errors when predicting the true values.

**Fig. 3:**
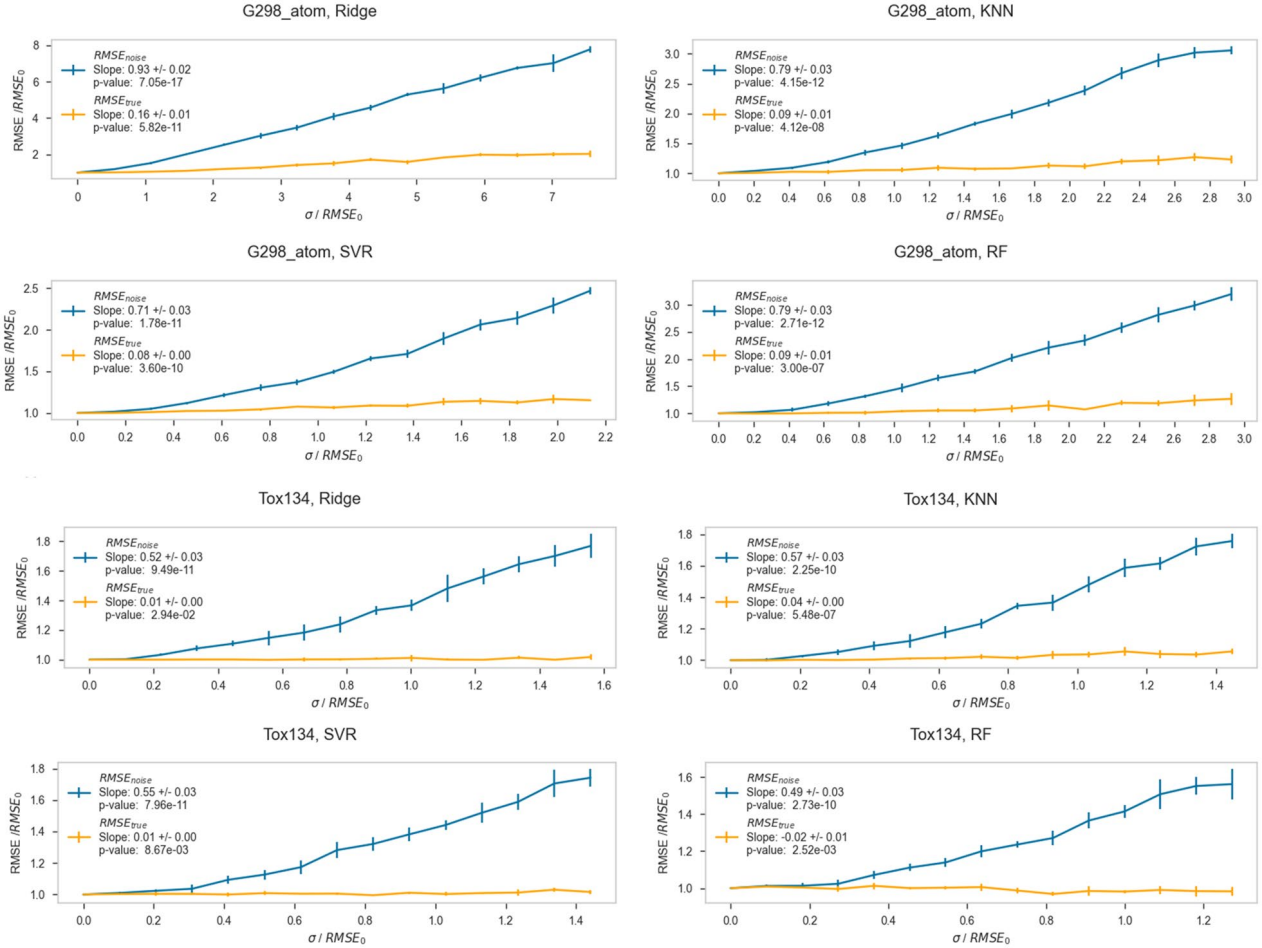
Plots showing RMSE versus the amount of random error added for the g298_atom and Tox134 datasets, for the Ridge regression, k-Nearest Neighbors (KNN), Support Vector Regression (SVR), and Random Forest (RF) algorithms. For each plot, the *y-axis* is RMSE, and the x-axis is the standard deviation (σ) of the Gaussian distribution of the added error. The blue lines show data evaluated on test sets with error, and orange lines show data evaluated on test sets without error

**Fig. 4:**
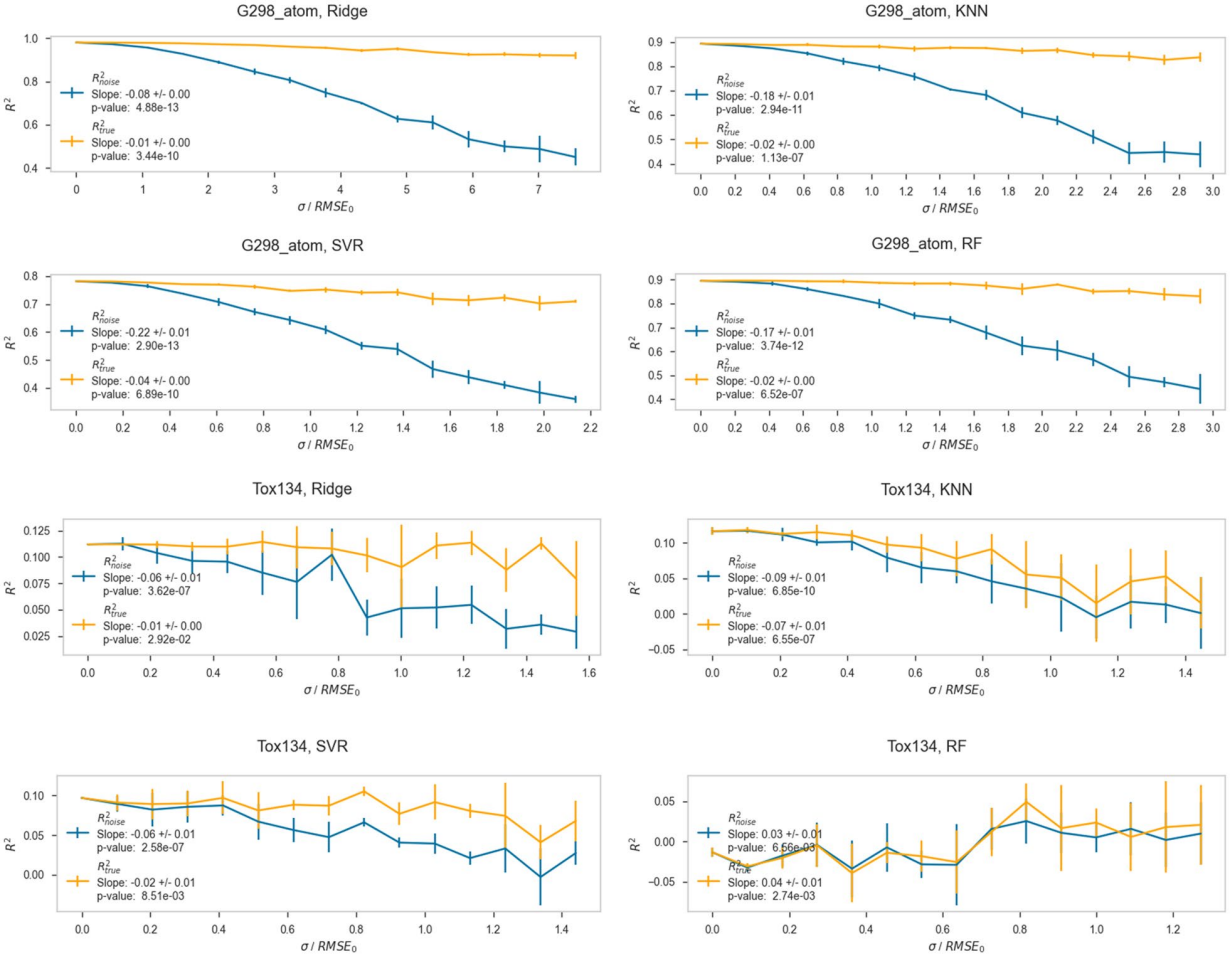
Plots showing R^2^ versus the amount of random error added for the g298_atom and Tox134 datasets, for the Ridge regression, k-Nearest Neighbors (KNN), Support Vector Regression (SVR), and Random Forest (RF) algorithms. For each plot, the *y*-axis is R^2^, and the *x*-axis is the standard deviation (σ) of the Gaussian distribution of the added error. The blue lines show data evaluated on test sets with error, and orange lines show data evaluated on test sets without error

The results for a representative datasets and algorithm are shown in Figs. 3 and 4 (additional figures for other datasets are available in the Additional file 1). In order to compare trends in data across algorithm and dataset, we chose to normalize the RMSE and the amount of added noise by RMSE_0_, which is the RMSE obtained from training and predicting on the original noiseless dataset. In the top subplot, the *y*-axis is RMSE/RMSE_0_. The *x*-axis is the standard deviation of the Gaussian distribution from which the added error was sampled (σ), divided by RMSE_0_. Therefore, the y-axis indicates the multiplicative increase in observed prediction error, while the x-axis is most accurately understood as the fractional amount of error inserted into the dataset standardized by the amount of prediction error seen in the noiseless dataset. In this figure, a constant value of 1 on the y-axis would corresponds to seeing the exact same error at a particular noise level as was seen when modeling the native dataset. Similarly, a line through the origin with a slope of 1 would represent the expected RMSE obtained if one compared Test_noise_ to Test_true_.

For each dataset and algorithm, the RMSE_noise_/RMSE_0_ clearly increases as σ/RMSE_0_ increases. The RMSE_true_/RMSE_0_ values increase slightly or stay essentially constant, depending on the dataset. What is qualitatively clear from these plots is that RMSE_true_/RMSE_0_ stays low and constant, while RMSE_noise_/RMSE_0_ rapidly outpaces it. These results, which investigate a variety of different datasets and endpoints, are consistent with the work of Cortés-Ciriano and coworkers, in which, for pIC_50_ datasets, the RMSE on the test set remained constant while noise was added to the training set [22]. The fact that RMSE_true_/RMSE_0_ remains nearly constant indicates these models are still accurately predicting the noiseless Test_true_ values despite being trained on increasingly noisy data in Train_noise_. The RMSE_noise_ being consistently higher than RMSE_true_ for each algorithm and dataset indicates that while the models are retaining their accuracy, our ability to validate the models as being accurate using Test_noise_ is significantly degraded. It is also clear that R^2^_noise_ and R^2^_true_ generally get worse with increasing σ/RMSE_0,_ and that R^2^_true_ is better than R^2^_noise_ for all noise levels. This trend is more apparent in dataset/algorithm combinations which have acceptably large R^2^ values, such as the quantum mechanical dataset G298_atom, than in datasets which have extremely low starting R^2^ values, such as the toxicological dataset Tox134. Especially with, for example, the combination of Tox134 and RF, both R^2^_noise_ and R^2^_true_ are 0, indicating that these predictions are not reliable. Having such a small R^2^ with a small dynamic range makes forming conclusions about this particular data tenuous at best.

To facilitate the comparison of these trends between algorithms and datasets, a representative quantitative measure of the observed behavior was chosen. First, the slope of RMSE_noise_/RMSE_0_ versus σ/RMSE_0_ (*m*_*noise*_) and the slope of RMSE_true_/RMSE_0_ versus σ/RMSE_0_ (*m*_*true*_) were obtained by fitting lines to the respective data. These slopes directly report on how RMSE_noise_ and RMSE_true_ behave with the addition of error to the dataset. For example, if *m*_*noise*_ is high, then RMSE_noise_ increases significantly as noise is added to the training set, meaning the algorithm becomes worse at predicting Test_noise_ as Train_noise_ becomes noisier. If *m*_*true*_ is high, then RMSE_true_ increases significantly as noise is added to the training set, meaning the algorithm is getting worse at predicting Test_true_ as noise is added. The ratio of *m*_*noise*_*/m*_*true*_ provides a single metric defining whether a model is predicting closer to true values or noisy values as the training set becomes noiser. If *m*_*noise*_*/m*_*true*_ is large, then RMSE_noise_ is increasing much faster than RMSE_true_, and the resultant models are predicting true values much more accurately than noisy values (as they should). This indicates our predictive power on noisy datasets using such an algorithm is likely much better than often perceived from our test/validation statistics. If *m*_*noise*_*/m*_*true*_ is close to 1, then RMSE_noise_ and RMSE_true_ are responding very similarly to noise, and the model is not predicting true values much better than noisy values. Table 3 shows *m*_*noise*_ and *m*_*true*_, and Table 4 shows *m*_*noise*_*/m*_*true*_ ratios.

**Table 3:** Slopes *m*_*noise*_ and *m*_*true*_ for each dataset and algorithm

Dataset	Slope	Ridge	kNN	SVR	RF
G298_atom	*m*_*noise*_	0.98 ± 0.011	0.79 ± 0.032	0.71 ± 0.032	0.79 ± 0.030
	*m*_*true*_	0.090 ± 0.010	0.09 ± 0.011	0.08 ± 0.00	0.09 ± 0.013
Alpha	*m*_*noise*_	0.79 ± 0.033	0.83 ± 0.037	0.87 ± 0.023	0.89 ± 0.032
	*m*_*true*_	0.12 ± 0.016	0.10 ± 0.012	0.12 ± 0.014	0.11 ± 0.013
Lip	*m*_*noise*_	0.40 ± 0.031	0.36 ± 0.024	0.44 ± 0.024	0.41 ± 0.031
	*m*_*true*_	0.020 ± 0.011	0.021 ± 0.010	0.062 ± 0.013	0.032 ± 0.012
Solv	*m*_*noise*_	0.75 ± 0.031	0.81 ± 0.031	0.89 ± 0.033	0.72 ± 0.031
	*m*_*true*_	0.13 ± 0.022	0.27 ± 0.011	0.27 ± 0.012	0.12 ± 0.012
BACE	*m*_*noise*_	0.52 ± 0.042	0.53 ± 0.041	0.67 ± 0.033	0.54 ± 0.031
	*m*_*true*_	0.041 ± 0.021	0.052 ± 0.011	0.23 ± 0.023	0.050 ± 0.011
Tox_102	*m*_*noise*_	0.44 ± 0.031	0.49 ± 0.043	0.44 ± 0.031	0.43 ± 0.031
	*m*_*true*_	0.010 ± 0.00	0.053 ± 0.011	0.00*^a^	0.010 ± 0.00
Tox_134	*m*_*noise*_	0.52 ± 0.034	0.57 ± 0.034	0.55 ± 0.031	0.49 ± 0.033
	*m*_*true*_	0.01*^a^	0.041 ± 0.00	0.010 ± 0.00	− 0.020 ± 0.010
LD_50_	*m*_*noise*_	0.44 ± 0.042	0.43 ± 0.042	0.48 ± 0.033	0.48 ± 0.031
	*m*_*true*_	0.00 ± 0.010	0.044 ± 0.016	0.083 ± 0.012	0.033 ± 0.012

^a^Entries marked with * have *p*-values above 0.05 and thus are not statistically significant

**Table 4:** Ratios of *m*_*noise*_*/m*_*true*_ for each dataset and algorithm

Dataset/algorithm	Ridge	kNN	SVR	RF
G_298_atom	11 ± 1.3	8.8 ± 1.4	8.9 ± 0.40	8.8 ± 1.6
Alpha	6.6 ± 1.1	8.3 ± 1.3	7.3 ± 1.0	8.1 ± 1.3
Lip	20 ± 12	17 ± 9.0	7.1 ± 1.9	13 ± 5.7
Solv	5.8 ± 1.2	3.0 ± 0.23	3.3 ± 0.26	6.0 ± 0.84
BACE	13 ± 7.7	10 ± 2.8	2.9 ± 0.44	11 ± 3.0
Tox_102	44 ± 3.1	9.2 ± 2.7	–^a^	43 ± 3.0
Tox_134	52* ± 3.1^b^	14 ± 0.84	55 ± 3.3	–^a^
LD_50_	–^a^	9.7 ± 4.4	5.8 ± 1.2	15 ± 6.3

^a^Entries marked with a—had a null or negative denominator

^b^Entries marked with an * are not statistically significant

Inspecting the values of *m*_*noise*_ and *m*_*true*_ in Table 3 reveals some consistent behaviors. For a given dataset, *m*_*noise*_ and *m*_*true*_ are reasonably constant across algorithms (across rows). This observation is consistent with the consistent behavior across algorithms that Cortés-Ciriano observed [22]. For a given algorithm, *m* (and to a lesser extent *m*_*true*_) vary more significantly over datasets (down columns). This indicates that the RMSE response to added error is consistent for a given dataset with different algorithms, and that the RMSE response is highly variable for a given algorithm across different datasets. These datasets were chosen specifically to encompass a range of experimental complexity and thus a range of native random experimental error. While not definite, the variable nature of the RMSE response to noise over datasets may indicate that these algorithms respond differently to different amounts of native error; Cortés-Ciriano and coworkers observed and commented on the differential response of algorithms to noise, but did not emphasize how noise response differed over different types of endpoints [22]. For example, the quantum mechanical datasets have high *m*_*noise*_ values (approaching 1) while toxicity datasets have more moderate slopes (near 0.5). It is expected that higher native error existed in the toxicological datasets compared to the quantum mechanical datasets and such error could have an impact in observing the effects of the additional simulated noise. This suggests that the RMSE response to additional noise likely decreases as the amount of native error in a dataset increases. In contrast, *m*_*true*_ varies little and does not follow a decreasing trend over datasets. This observation indicates that these algorithms are capable of finding the “true” values as simulated error was added, regardless of the amount of native error in the original dataset.

Analyzing the *m*_*noise*_*/m*_*true*_ ratios in Table 4 reveals how the relative noise responses of RMSE_noise_ and RMSE_true_ change across algorithm and dataset. One immediate observation is that the ratios for the Tox102 and Tox134 datasets are more highly variable than the ratios of the other datasets. This variability comes from the fact that *m*_*true*_ is generally very small, so small changes in this small number lead to large fluctuations in the *m*_*noise*_*/m*_*true*_ ratios. This instability could be viewed as one detriment of this metric. It is also apparent that the Tox102 and Tox134 datasets have the highest ratios, albeit with large variability. This means that as noise is added to these datasets, RMSE_noise_ increases much more rapidly than RMSE_true_, and the algorithms can predict the true values more accurately than the noisy values. We expect that Tox102 and Tox134 have relatively high native error compared to the quantum mechanical, physiochemical, and biochemical datasets, and we propose that the addition of more error to these datasets does not affect the algorithms ability to predict the true values as drastically as it does to the other datasets. This proposal is supported by the fact that the *m*_*true*_ values for Tox102 and Tox134 are roughly an order of magnitude smaller than *m*_*true*_ values for the G298_atom, Alpha, and Solv datasets.

These experiments used PCA to reduce the number of descriptors involved in prediction while maintaining as much variance as possible. Using PCA achieves dimension reduction by forming linear combinations of the original descriptors; although this process ultimately reduces the physical interpretability of the model, it does provide a significant computational advantage because the predictive algorithm has fewer, but more information dense, variables to work with. However, given that this preprocessing step is somewhat uncommon in the QSAR literature, the effect of using PCA on the *m*_*noise*_/*m*_*true*_ ratios was tested. The results without PCA in Table 5 show mixed trends when compared with Table 4. The most dramatic effect is seen across each dataset using the Ridge algorithm, for which the ratios all drop significantly. This is expected because Ridge regression uses a regularization to mitigate variance at the expense of adding bias; this means that the algorithm is sensitive to having many feature variables that complicate finding a useful trend. Therefore, when PCA is not used, the Ridge algorithm does not predict the true values as well and the ratio decreases. For kNN and SVR however, the ratios are not sensitive to the use of PCA. This experiment was not carried out for RF because computational time scales with the number of descriptors, so performing the workflow without dimension reduction made the calculation time unreasonable. The other apparent trend is that the ratios for the Tox102 and Tox134 datasets are significantly reduced without PCA. This result suggests that predicting the true values in these datasets is sensitive to the number of descriptors, so that when many extraneous descriptors are used the ratios become smaller.

**Table 5:** Ratios of *m*_*noise*_/*m*_*true*_ without Principal Component Analysis

Dataset/algorithm	Ridge	kNN	SVR
G_298_atom	1.4 ± 0.10	8.0 ± 1.4	5.1 ± 0.22
Alpha	1.7 ± 0.13	13 ± 3.4	4.7 ± 0.58
Lip	1.9 ± 0.53	12 ± 5.0	3.1 ± 0.26
Solv	1.4 ± 0.080	2.5 ± 0.24	3.3 ± 0.48
BACE	1.6 ± 0.10	6.8 ± 0.95	14 ± 0.64
Tox_102	1.5 ± 0.075	6.4 ± 0.70	7.6 ± 0.29
Tox_134	1.0 ± 0.15	7.8 ± 1.2	10 ± 0.44
LD_50_	1.3 ± 0.15	7.5 ± 1.0	31 ± 1.7

Additionally, it is useful to contextualize the amount of simulated error which has been added to these datasets within what is known about experimental uncertainties. Estimates for most of the endpoints used in this study are not readily available, however Kramer, Kalliokoski and colleagues found from an examination of the ChemBL database that heterogeneous pIC_50_ data has an average standard deviation of 0.68 log units [19]. For the BACE dataset, which uses a pIC_50_ endpoint, 1.1 log units of noise were added, or 1.6 times the average standard deviation reported in ChemBL.

#### Gaussian process results

In addition to quantifying how *accurate* QSAR predictions are, it is very useful to quantify how *precise* predictions are. While machine learning algorithms such as Ridge regression, kNN, SVR, and RF do not have a direct method of quantifying the precision or uncertainty of the predictions made on each molecule, the Gaussian Process (GP) algorithm does provide direct uncertainties for each of its predictions. We utilized the GP algorithm to investigate how prediction precision is affected by the addition of simulated error into each dataset.

There has been extensive work carried out on the general topic of prediction uncertainties in the QSAR literature, typically involving Bayesian methods. Wood and coworkers analyzed model output with the Kullback–Leibler divergence to generate estimates of prediction uncertainty [55]. Burden introduced GP to the QSAR community [56], Obrezanova and coworkers later applied GP to ADME properties, highlighting its usefulness as an application in drug discovery [57, 58], and many other works have utilized GP with other endpoints [59–61]. Cortés-Ciriano and coworkers applied GP to the field of proteochemometrics, using the prediction uncertainty to estimate the applicability domain of the model [62]. Conformal prediction is a non-Bayesian technique which also produces confidence intervals, and has been applied often in QSAR and computational toxicology [63–71]. Conformal prediction has the advantage that it does not require the selection of a prior distribution like GP, which means that no assumptions need to be made about the underlying distribution of the data. While a quantitative comparison of conformal prediction and GP is outside the scope of this work, the comparison has been made elsewhere [72]. The advantage of providing prediction uncertainties in the field of QSAR motivated the study of GP in this work, in order to understand how the addition of noise to the various datasets affects the precision of the predictions.

Following the analysis of the Results section for the other algorithms, we examined RMSE and R^2^ for GP to give a measure of prediction accuracy. However, to quantify prediction precision, we examined the prediction uncertainty σ_ŷ_, or width of each individual prediction. We examined both the mean σ_ŷ_ (Eq. 21), which is the average of all the individual prediction uncertainties, and the σ_ŷ_ 95% confidence interval (Eq. 22), which is the spread of the individual prediction uncertainties. An important point of emphasis is that the prediction uncertainty σ_ŷ_ is completely dependent on the descriptor values and is independent of whether the prediction is evaluated using the true test set or the noisy test set. In other words, the precision of a prediction is completely dependent on how close that molecule is in feature space to other molecules. This behavior contrasts with the metrics RMSE and R^2^, which depend entirely on whether the “true” answer comes from a true test set or a noisy test set.

The GP algorithm also has the option to include information about the uncertainty of the experimental measurement vector *Y;* we will define this uncertainty vector as *σ*_*y*_ (Eq. 24). When *σ*_*y*_ is given to GP, the algorithm can incorporate this information to adjust the precision of each element in its prediction vector *Ŷ*. Ignoring native error in the datasets, the uncertainty in the measurements *σ*_*y*_ is just the width of the gaussian distribution from which the error was sampled; each term *σ*_*yn*_ within the vector *σ*_*y*_ will be the same.

19 $${\hat{\text{Y}}} = {\hat{\text{y}}}_{1} , {\hat{\text{y}}}_{2} , \ldots , {\hat{\text{y}}}_{n}$$

20 $$\sigma_{{{\hat{\text{y}}}}} = \sigma_{{{\hat{\text{y}}}1}} , \sigma_{{{\hat{\text{y}}}2}} , \ldots , \sigma_{{{\hat{\text{y}}}n}}$$

21 $$Mean \sigma_{{{\hat{\text{y}}}}} = \frac{1}{n}\mathop \sum \limits_{i = 1}^{n} \sigma_{i}$$

22 $$\sigma_{{{\hat{\text{y}}}}} 95\% CI = \frac{1.960}{{\sqrt n }}\left[ {\frac{1}{n}\mathop \sum \limits_{i = 1}^{n} \left( {\sigma_{i} - Mean \sigma_{{{\hat{\text{y}}}}} } \right)^{2} } \right]$$

23 $$Y = y_{1} , y_{2} , \ldots , y_{n}$$

24 $$\sigma_{y} = \sigma_{y1} , \sigma_{y2} , \ldots , \sigma_{yn}$$

**Fig. 5:**
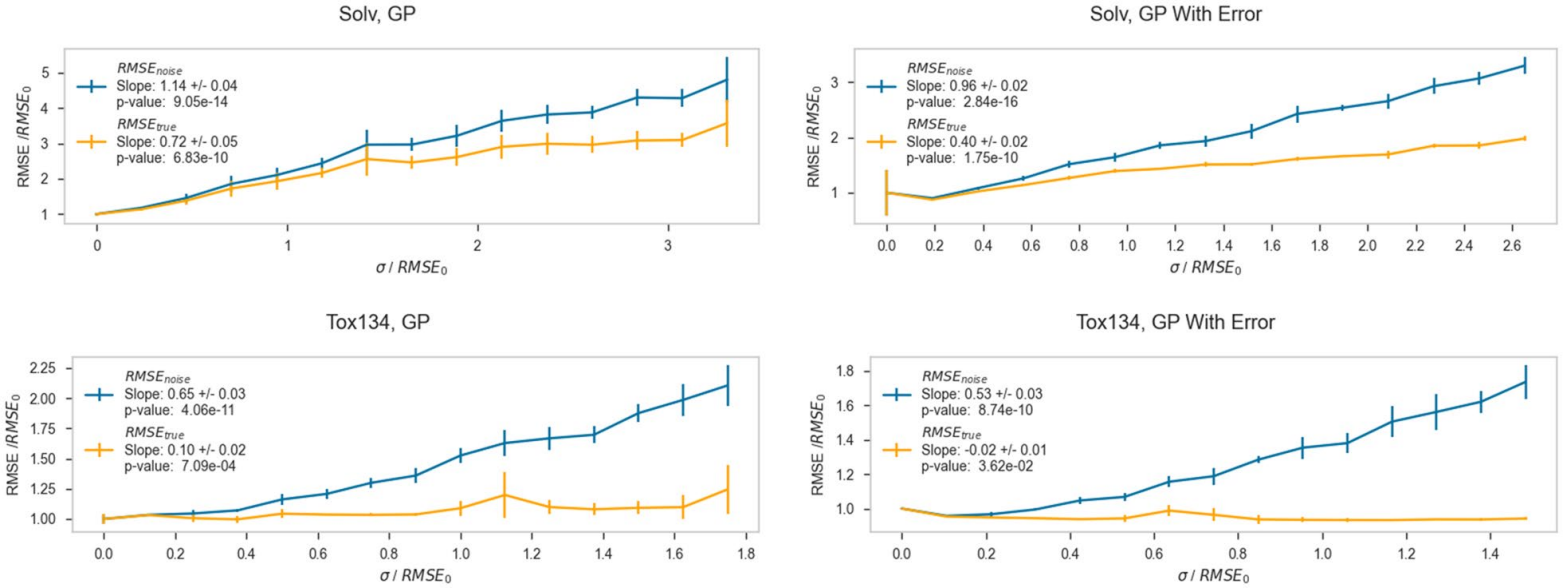
Plots showing RMSE versus the amount of added error to the Solv and Tox134 datasets, using Gaussian Process algorithm. Plots on the left are for data where no information about the added error *has not* been fed to the GP algorithm, and plots on the right are for data where added error information *has* been fed to the GP algorithm

Plots of a selection of GP results are shown in Fig. 5, and the tabulated GP prediction accuracy results are shown in Table 6. Each row gives the ratio of *m*_*noise*_ to *m*_*true*_ for each dataset. The first column shows values for which uncertainty in the *Y* vector was not provided, and the second column shows values for which uncertainty in the *Y* vector was provided. For the first column, without *σ*_*y*_, the *m*_*noise*_*/m*_*true*_ ratios are all greater than 1, however they are somewhat lower than average than the ratios for the other algorithms, which are shown in Table 4. This result suggests that GP when instructed to assume no uncertainty exists in its training set is not as robust to error inserted into its training set, at least in comparison to the other algorithms in this study. However, in the second column, where *σ*_*y*_ was provided to GP, the ratios increased. The *m*_*noise*_*/m*_*true*_ ratio approximately doubled for the BACE dataset, and more than quadrupled for the Alpha dataset. This result shows that when information about the uncertainty in the measurements is known, the GP algorithm makes predictions which are much closer to the true values than the artificially noisy values with a similar robustness to other learning algorithms. The ability to directly incorporate measurement uncertainty into predictions is a unique feature of the GP algorithm and provides useful insight into how prediction accuracy and precision are connected to experimental error.

**Table 6:** Ratios of *m* to *m*_*true*_ for the Gaussian Process algorithm

Dataset	No σ_y_	With σ_y_
G_298_atom	6.9 ± 1.5	1.7 ± 0.26
Alpha	1.8 ± 0.11	9.3 ± 0.37^a^
Solv	1.6 ± 0.24	2.4 ± 0.17^a^
BACE	3.7 ± 2.0	8.6 ± 1.6^a^
Tox_102	2.7 ± 1.7	–^b^
Tox_134	6.5 ± 1.6	–^b^
LD_50_	5.3 ± 0.74	5.5 ± 0.83

^a^Slopes *m*_*noise*_ and *m*_*true*_ were calculated excluding the first point due to a discontinuity in the line

^b^The slope *m*_*true*_ was negative for these plots, so the slope ratio was not calculated

As mentioned above, GP provides quantitative information about the uncertainty in its predictions, which is contained in the vector σ_ŷ_. In order to investigate how prediction precision is affected by the addition of simulated error, the prediction uncertainty *σ*_*ŷ*_ was plotted versus the amount of added error *σ*. Additionally, the effect of providing measurement uncertainty *σ*_*y*_ to the GP algorithm was explored. Table 7 shows the slopes of mean *σ*_*ŷ*_ versus *σ* and the *σ*_*ŷ*_ 95% confidence interval versus *σ*, for GP models where *σ*_*y*_ was and was not provided. Slopes were obtained by linear fits to the data, although in some cases the data was significantly non-linear; while this analysis is imperfect, we feel that it still allows useful qualitative trends to be captured.

**Table 7:** Slopes of mean σ_ŷ_ and σ_ŷ_ 95% CI versus σ for the Gaussian Process algorithm. Results are shown with and without the input of σ_y_ into the algorithm

Dataset	No σ_y_	No σ_y_	With σ_y_	With σ_y_
	Mean σ_y_	σ_y_ 95% CI	Mean σ_y_	σ_y_ 95% CI
G_298_atom	1.0	0.40	0.52	− 0.10
Alpha	1.1	0.16	0.44^a^	0.32^a^
Solv	0.94	− 0.19	0.10	0.10
BACE	0.25	0.38	− 0.12	− 0.35
Tox_102	0.32	0.028	− 0.96	− 0.48
Tox_134	0.49	0.53	− 0.66	− 0.17
LD_50_	0.66	− 0.39	− 0.60	0.14

^a^The first point was omitted in these calculations because of a discontinuity in the line

**Fig. 6:**
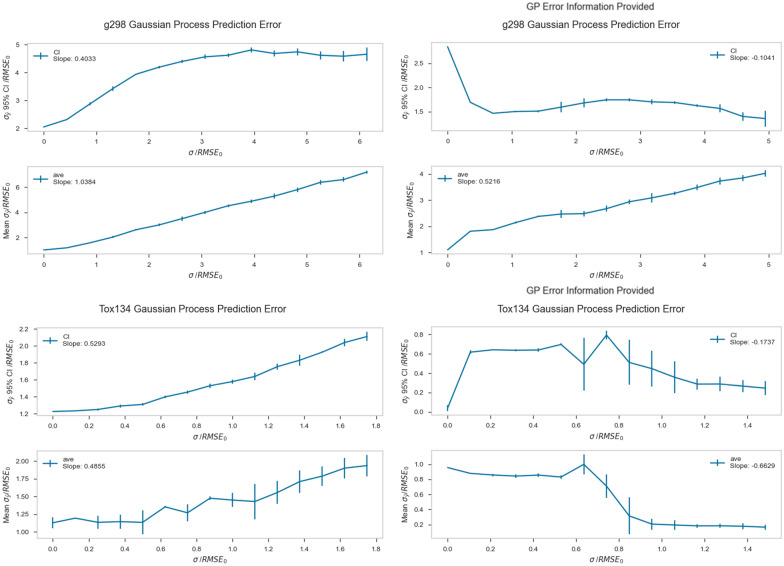
Plots showing prediction error versus amount of added error to the G298_atom and Tox134 datasets, using the Gaussian Process algorithm. The top subplots show the average 95% confidence interval for prediction error versus added error. The bottom subplots show the mean prediction error versus amount of added error

Inspection of Table 7 and the accompanying Fig. 6 show that when the measurement uncertainty *σ*_*y*_ is withheld from the algorithm, the slopes of mean *σ*_*ŷ*_ versus σ are all positive. This indicates that prediction precision gets worse as noise is added into the data. These slopes also generally become smaller as the qualitative complexity of the datasets increase. This could be attributed to the amount of native error present in each dataset. For example, while the G298atom dataset has no experimental uncertainty because it is composed of quantum mechanical endpoints, the Tox102 dataset is composed of in vitro measurements with a large degree of variability. Because the Tox102 dataset contains more native error, the prediction precision is not as sensitive to the addition of noise.

The slope of prediction uncertainty *σ*_*ŷ*_ is very sensitive to the inclusion of measurement uncertainty *σ*_*y*_. Including measurement uncertainty in the calculation decreases the slope for each of the datasets, even causing some of the slopes to become negative. This indicates that information about the variability in the measurements reduces the effect that added error has on the prediction precision. This reductive effect is mild for the quantum mechanical and physiochemical datasets but becomes more pronounced for the in vitro and in vivo datasets. This result shows that even when datasets have large uncertainty in the measurements, the predictions from GP can apparently become *more precise* as more error is introduced as long as the magnitude of that error is known, the error is normally distributed, and the error is provided as an input. Error in datasets is not always known, nor is it always normally distributed. The experiments described here nevertheless provide a foundation for understanding how the effect of added error can be mitigated when using Gaussian Processes, when the nature of that error is known. Because of the nature of this experiment, the distribution and magnitude of the error was predetermined, which, admittedly, is not a situation that is common in QSAR modeling.

The 95% confidence interval of *σ*_*ŷ*_ shows more complicated behavior as error is added to the datasets. When measurement uncertainty is withheld from the algorithm, the slope of 95% confidence interval versus σ is positive for each dataset except Solv and LD_50_, which show negative trends. Additionally, the G298atom and Alpha datasets show a quadratic trend which levels off at high values of *σ*, which contrasts with the more linear trends observed in the other datasets. This indicates that, generally, the distribution of prediction error is getting larger as more error is added to the datasets. In other words, as more error is added to the datasets, not only does the average prediction uncertainty increase, but the spread in those average uncertainties becomes larger as well. It remains unclear why this behavior is different for the Solv and Tox134 datasets. Although the Solv dataset shows a relatively flat slope, the LD_50_ dataset shows a clearly negative trend.

When measurement uncertainty is provided to the GP algorithm, the trends in the 95% confidence interval of *σ*_*ŷ*_ change. The change in behavior is inconsistent and complicated across the datasets but including information about measurement uncertainty clearly affects the trends significantly. One consistent effect is that the error bars become much smaller, which shows that the results are much more tightly distributed between the 5 repetitions at each level of *σ* added to the datasets.

Additionally, it is possible to evaluate the mean prediction uncertainty that GP provides by comparing it to the mean experimental estimate of uncertainty for pIC_50_ provided by Kolliokoski and colleagues [19]. The mean σ_ŷ_ can be obtained using RMSE_0_ and σ_noise_ of 0 for the GP calculations on the BACE dataset. Using the RMSE_0_ value of 0.98 for the GP calculations on the BACE dataset, the mean σ_ŷ_ is 0.79 log units. The estimated experimental uncertainty for pIC50 is 0.68 log units, so GP’s prediction uncertainty is 1.2 times the experimental estimate, when no simulated noise has been added to the dataset.

## Discussion and conclusions

The purpose of this work is to examine the common assumption that QSAR models cannot make predictions which are more accurate than their training data. Many other works have contributed to this general topic, including thorough estimations of the random error in K_i_, [20] IC_50_, [19] and cytotoxicity [21] databases and an investigation of the noise tolerance of machine learning algorithms with IC_50_ data [22]. These works and others have supported the well-known phenomenon that machine learning algorithms are generally tolerant to noise. There is a general contention however that experimental uncertainty sets the upper limit of in silico predictions [20], and this study has attempted to examine that assertion. This work has attempted to ask, in the presence of increasingly noisy data, if these algorithms can formulate a trend that predicts closer to the true values than the artificial noisy values. However, investigation of this central hypothesis has two main limitations. The first limitation is statistical, which is that experimental values are typically only single values. When multiple values are available, there are still too few to reliably approximate the population mean for the measurement. This means that QSAR models are built on data which may poorly capture the physical reality of the trends being modeled. This limitation is recognized by the field, but there is little that can be done without increasing the rate of experimentation. The second limitation is the assumption that test sets and validation sets have no associated error, or at least this assumption is necessitated by the methods used. Because QSAR models are evaluated on these test and validation sets, this means that QSAR models are being judged by their ability to predict error laden values, when they should be judged by their ability to predict the population means of measurements. The result of these limitations is that it is commonly assumed/stated that QSAR models cannot make predictions which are “better” or more accurate than their training data. A more exact statement would be that cross/external validation statistics (our standard metrics of predictivity) for QSAR models are limited based on the accuracy of the dataset. The present work has designed a set of experiments to examine these limitations and this hypothesis by adding simulated error into a variety of representative QSAR datasets and designating two classes of test sets. The first class of test set comes from “true” error free values, and the second class of test set comes from the “noisy” error laden values. The difference in performance metrics between these two classes of test sets allows us to examine whether models can really generate predictions which are more accurate than the noisy data they were trained on. The error added to the datasets in this work was Gaussian distributed, which provides a convenient analogy for real-world data situations in which endpoint values fall somewhere on a Gaussian distribution of error. It is true that this situation is not always the case. Despite the fact that the present experiments are testing a hypothesis that could be labeled an “ideal” case of dataset error, we posit that it still provides useful conclusions that have not been clearly stated in QSAR modeling literature.

The results show that there is a consistent difference in the RMSE when predictions are evaluated against the true and noisy test sets, across 5 algorithms and 8 datasets. The RMSE_true_ values are all lower than the corresponding RMSE values. When increasing amounts of error were added to the datasets, the difference between RMSE_true_ and RMSE became larger. This indicates that these models are predicting true values more accurately than noisy values, even when the algorithms are trained on data with large amounts of added simulated error. This scenario mirrors what likely happens for many QSAR models. A model is built on data with an unknown amount of error, which means that each experimental value may fall an unknown distance away from the true population mean for that measurement. Evaluation statistics for the QSAR model are then generated on internal test sets or an external validation set which are composed of values with unknown amounts of error. The RMSE, when calculated for these test sets, may be quite high, and thus the model is judged to be flawed. Work examining uncertainty in pK_i_ data asserts that if the uncertainty in training and validation sets are comparable, then the minimum RMSE obtainable should be equivalent to the uncertainty in the experimental data [20]. While this applies to situations in which experimental uncertainty estimates are available, it does not as readily apply when these estimations are unavailable. These results show that those models may very well be predicting the population means of those measurements, but this fact is obscured by the error in the test sets. Even from a very conservative interpretation of the results shown here, this study indicates that this situation is plausible.

The results also show that the difference between the observed RMSE and the unknown RMSE_true_ depends on algorithm and dataset complexity. This is an important observation, because it suggests that when models using different algorithms are compared, they may have significantly different accuracies, even if the observed RMSEs are very close. For example, examining the Solv row in Table 3, the *m*_*noise*_*/m*_*true*_ ratio is 3.3 for SVR and 6.1 for RF. This means that in a real modeling situation, if these SVR and RF algorithms produced the same RMSE for the Solv dataset, the RMSE_true_’s (and the relevant comparison) would be different by a factor of 1.8. Because real world datasets are undeniably rife with unknown amounts of error, this example demonstrates that comparing QSAR models through error laden test sets may be producing misleading conclusions in terms of model performance.

It is important to recognize that error in training sets appears to result in only a minor increase in “true” predictive error as assumed in this work (at least when work with datasets containing 1000 datapoints). In general, QSAR evaluation techniques cause us to perceive large amounts of predictive error when our training sets have error; this phenomenon is represented by the large RMSE_noise_ (what is observable in the general case) compared to the small RMSE_true_ (what unobservable in the general case). These observations were made by Cortés-Ciriano and coworkers on pIC_50_ datasets, and the current work complements and extends those initial studies [22]. Therefore, new learning methods will not resolve the issue. While some methods like Gaussian Processes and Conformal Prediction take error into account as part of training and allow modelers to estimate prediction precision, there are associated limitations. Conformal Prediction requires that a segment of the training set be put aside for calibration, while Gaussian Process requires a reasonable prior distribution and some knowledge of the experimental uncertainty to be effective. Much effort has been given towards analyzing experimental uncertainties for endpoints such as pK_i_, [20] pIC_50_, [19] and cytotoxicity [21] using public databases, providing useful inputs for methods like Gaussian Process and Conformal Prediction. Efforts towards estimating uncertainties of other common QSAR endpoints would be welcome.

## Supplementary Information

**Additional file 1.** Information about datasets and supplemental plots can be found in the additional file.

## Data Availability

All code used in this work, as well as the datasets containing SMILES strings, QSAR ready SMILES strings, and descriptor values, can be found in our public GitHub repository here: https://github.com/USEPA/CompTox-ChemInf-ModelExperiments-ErrorEffects/tree/SIRepo. All the necessary information to inspect the code and reproduce the results in this work can be found in the repository.
