# Supplementary material for: The effect of noise on the predictive limit of QSAR models
Source: J Cheminform. 2021 Nov 25;13:92. doi: 10.1186/s13321-021-00571-7 (PMC8613965; doi:10.1186/s13321-021-00571-7)
Supplement: Supplementary file 1 — Additional file 1. Information about datasets and supplemental plots can be found in the additional file. [file 13321_2021_571_MOESM1_ESM.docx]

Additional file 1

The Effect of Noise on the Predictive Limit of QSAR Models

*Scott S. Kolmar* and Christopher M. Grulke*

US Environmental Protection Agency, Office of Research and Development, Center for Computational Toxicology and Exposure, Research Triangle Park, NC, USA

# Sci. entific Code

The scientific code used to generate the results for this project was written in Python 3.6. The major Python libraries used include scikit-learn, pandas, numpy, matplotlib, pickle, and padelpy. All the function used to write the code, as well as a README file containing directions on how to reproduce the results, can be found in a public US Environmental Protection Agency (EPA) Github repository here: <https://github.com/USEPA/CompTox-ChemInf-ModelExperiments-ErrorEffects/tree/SIRepo>

# Datasets

## S2.1. Data Sources

The datasets G298_atom, Alpha, Lip, Solv, and BACE were taken from MoleculeNet,^1^ which offers several curated datasets for benchmarking purposes in the field of cheminformatics and QSAR. Details about the specific datasets taken from this collection are described below, followed by the details of the remaining datasets.

The quantum mechanical data used in this study is composed of 134,000 organic molecules with up to 9 heavy atoms, and the endpoints were calculated using DFT (B3LYP/6-31G(2df,p). ^2, 3^ This data is split into two separate datasets by endpoint. The first endpoint (G298_atom) is the atomatization free energy at 298.15 K, ΔG^o^_at_ (kcal mol^-1^). The second endpoint (Alpha) is the isotropic polarizability, α (Bohr^3^).

Two physiochemical datasets are used in this study. The first is a lipophilicity dataset (Lip) composed of 4,200 molecules, and the endpoint is the experimentally measured octanol/water distribution coefficient measured at pH = 7.4, logD.^4^ The second is a solvation dataset (Solv) composed of 642 molecules, and the endpoint is the experimentally measured hydration free energy, ΔG^o^_hyd_ (kcal mol^-1^).^5^

The biochemical dataset used in this study is the human β -secretase-1 dataset (BACE) which is composed of 1,513 molecules, and the endpoint is the experimentally measured pIC_50_ value for each molecule acting as an inhibitor for human β -secretase-1. ^6^

There are two *in vitro* toxicological datasets used in this work. Both toxicological datasets were taken from the United States Environmental Protection Agency’s (EPA) Toxcast^7, 8^ invitro_db_v3 database, and they both have endpoints associated with molecule binding to the peroxisome proliferator-activated receptor gamma (PPAR-gamma).^9, 10^ The first is the Tox102 dataset, which is composed of 971 molecules, and the endpoint is the logAC_50_ value PPAR-gamma binding via a *cis* activation assay. The second is the Tox134 dataset, which is composed of 1,347 molecules, and the endpoint is the logAC_50_ value PPAR-gamma binding via a *trans* activation assay.

The *in vivo* toxicological dataset LD50 is composed of 5,003 molecules, and the endpoint is the logLD_50_ (mg kg_bw_^-1^) value for rat acute oral toxicity. Values in this dataset were assembled from various databases as described in reference 5, but over 75% of the data was curated through the EPA’s DSSTox database.

## S2.2. Descriptor Generation and Data Used in this Work

The databases were used to obtain endpoint data and to obtain molecule identifiers, which were typically smiles strings. These smiles strings were passed through either the OPERA software^11^ or the python library Padelpy to generate Padel descriptors. The OPERA software automatically converts smiles strings to “QSAR ready smiles” as described in reference 11. When Padelpy was used to generate descriptors, the OPERA software was utilized to convert smiles strings to QSAR ready smiles first. Only 2D Padel descriptors were used. Occasionally, null or infinite values were produced for some descriptor values, for some molecules. When this occurred, columns (descriptors) which contained these values were dropped for the entire dataset; this task is written into code provided in S1.

Each of the datasets which was used to generate the results, complete with 1,444 Padel descriptors, endpoint, molecule identifier unique to each dataset, and smiles string, is provided in the Github account provided in S1. The datasets are stored as CSV files and are easily converted into Pandas dataframes.

##

## S2.3. Data Distributions

**Figure S1**. Box and whisker plots and violin plots for the G298_atom, Alpha, Lip, and Solv datasets. On the box and whisker plots, the boxes show the interquartile range, the black bar is the median, the whiskers show the range, and outliers are shown beyond the range. For the violin plots, a miniature box and whisker plot with identical features can be seen in black, while the orange represents the estimated distribution function of the data.


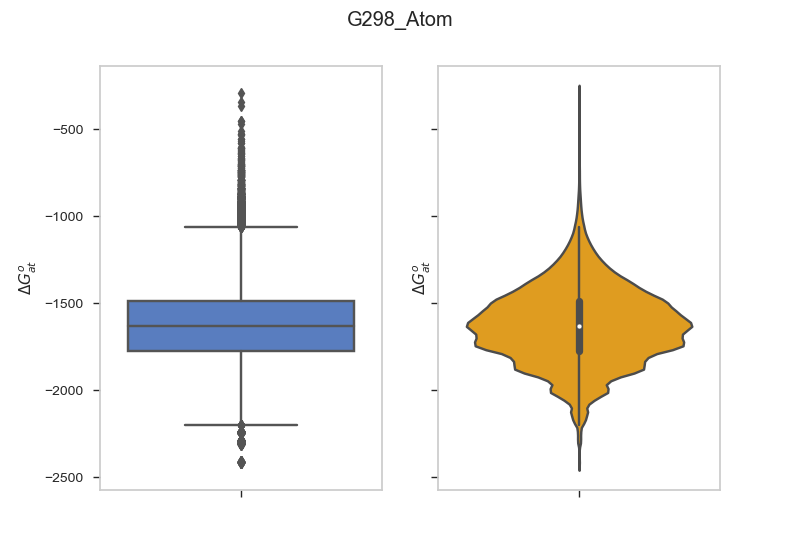

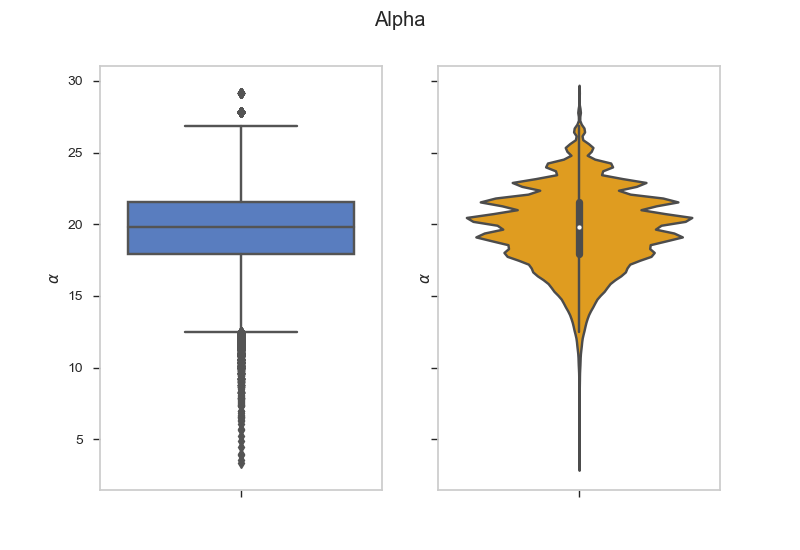

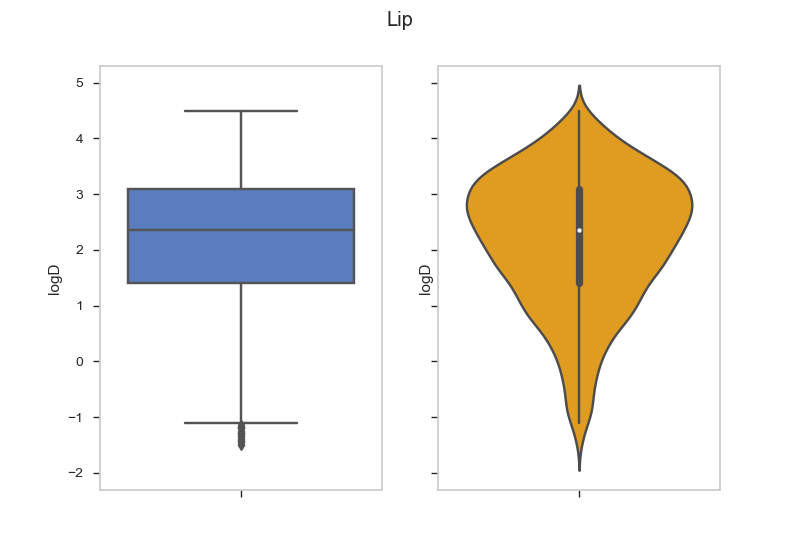

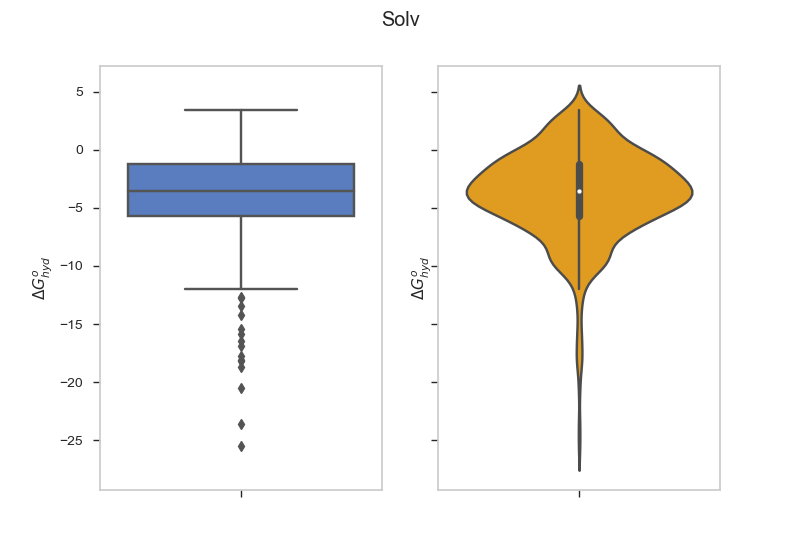


**Figure S2.** Box and whisker plots and violin plots for the BACE, Tox102, Tox134, and LD_50_ datasets. On the box and whisker plots, the boxes show the interquartile range, the black bar is the median, the whiskers show the range, and outliers are shown beyond the range. For the violin plots, a miniature box and whisker plot with identical features can be seen in black, while the orange represents the estimated distribution function of the data.


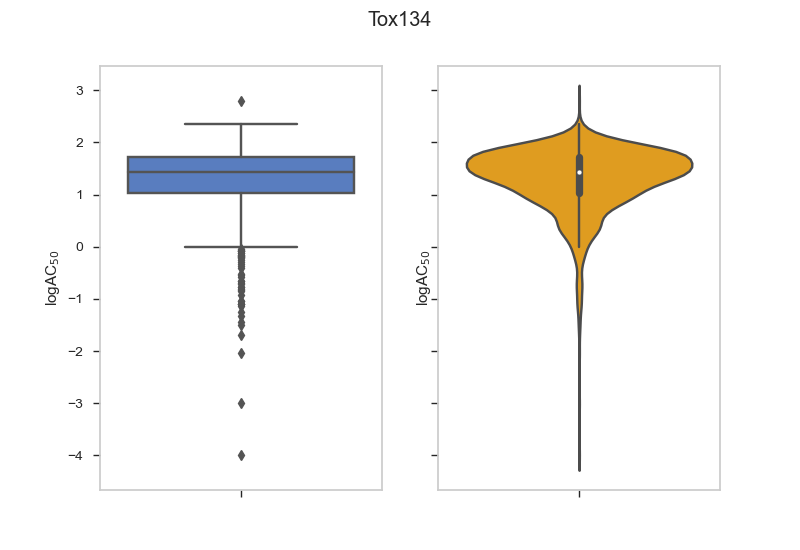

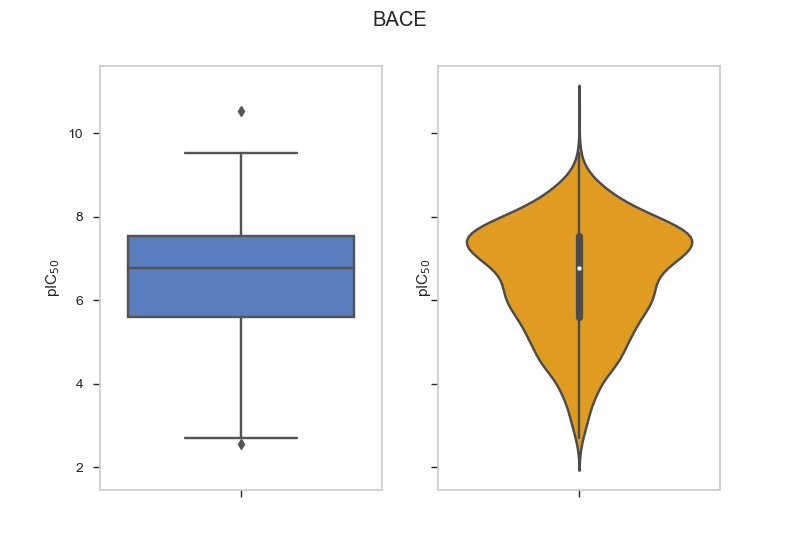

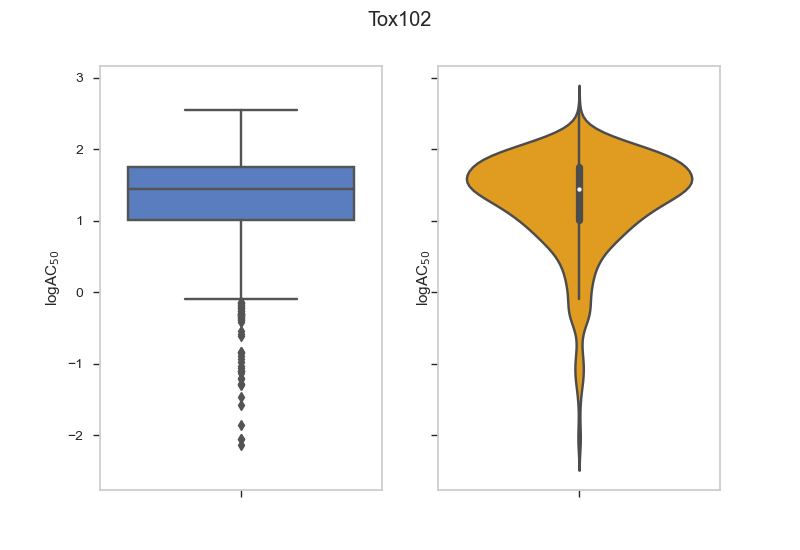

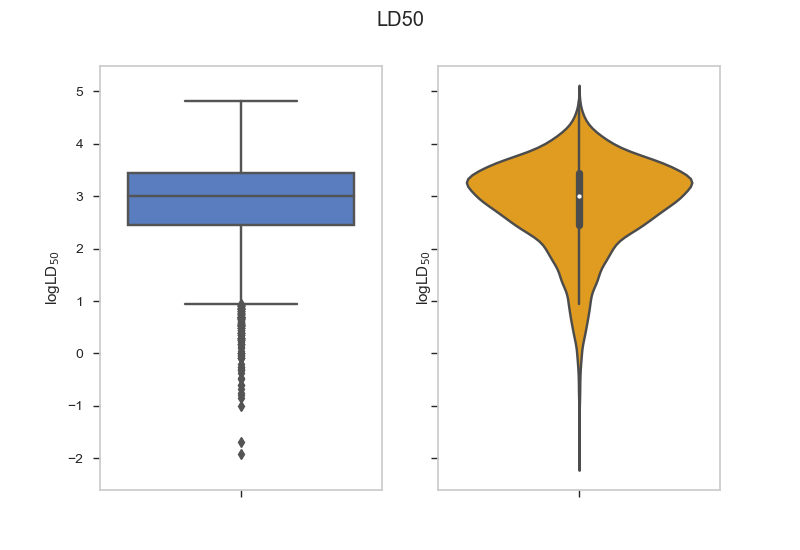


# **S.3.** Figures

## **S3.1.** RMSE and R^2^ Plots


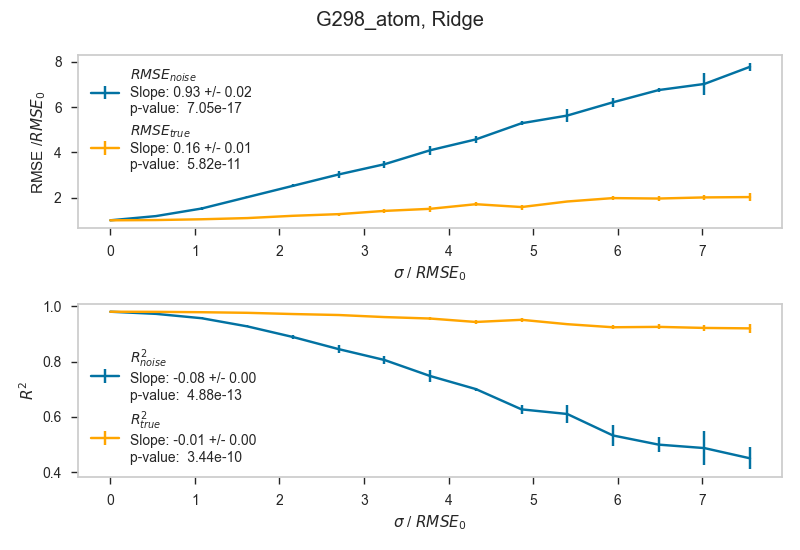

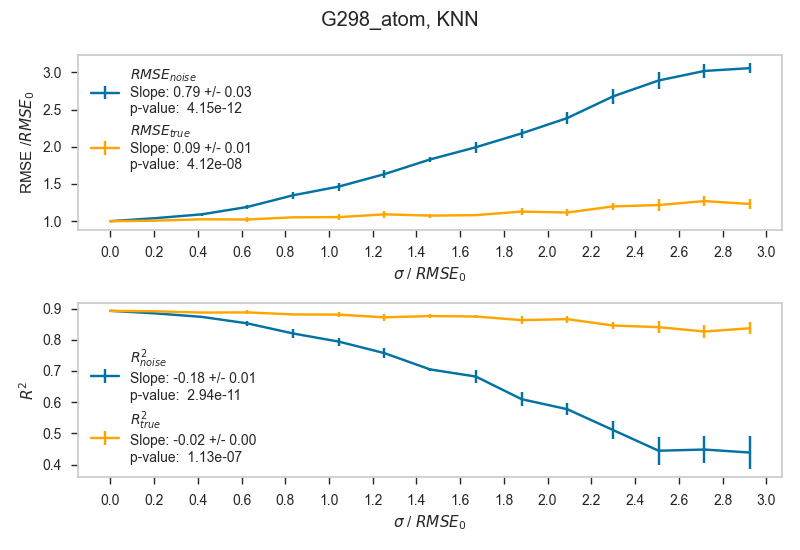

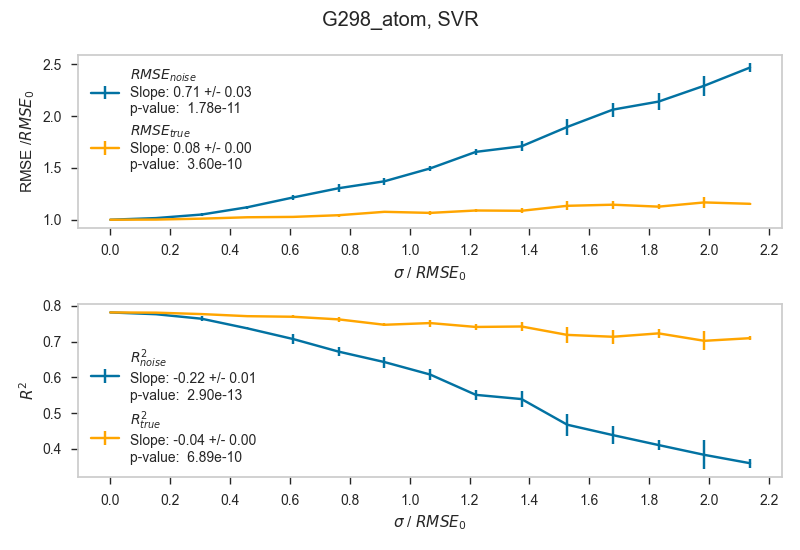

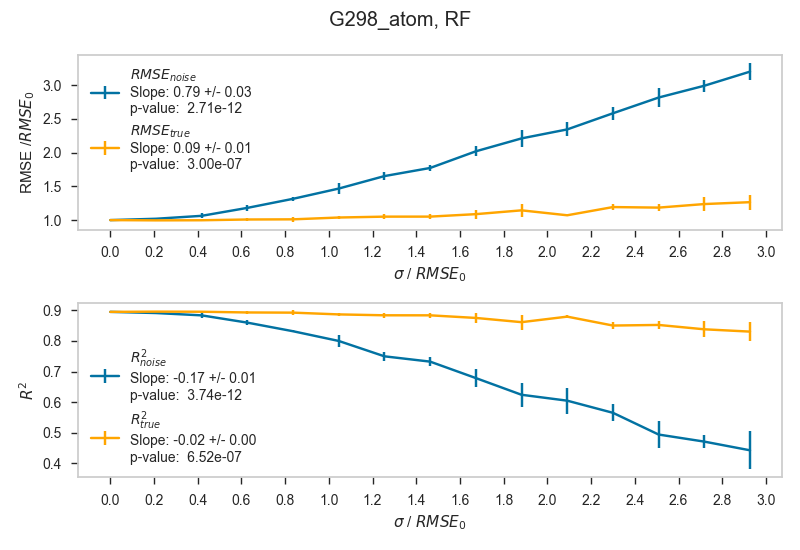


**Figure S3**. Plots showing RMSE, RMSE_true_, R^2^, and R^2^_true_ as functions of the standard deviation (σ) of the Gaussian distribution of the added random error for the G298_atom dataset. Each plot is a separate algorithm. Blue lines represent metrics for predictions which are evaluated against Test_noise_, and orange lines represent metrics for predictions which are evaluated against Test_true_.


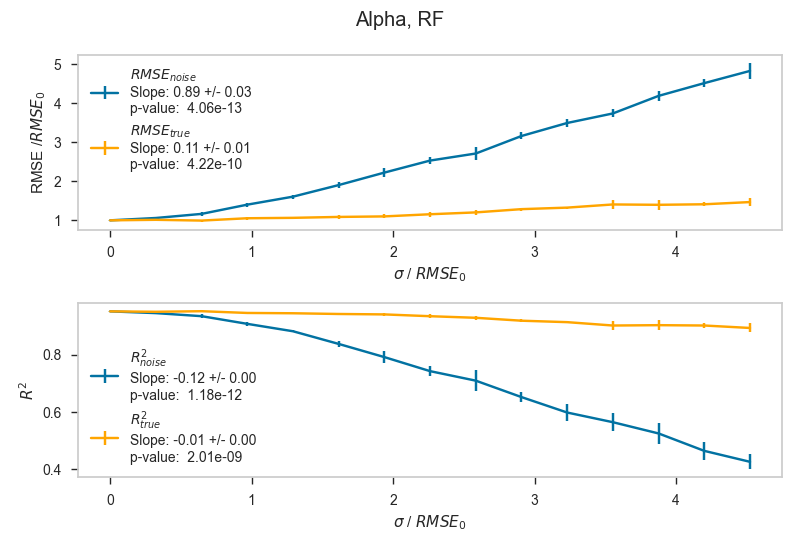

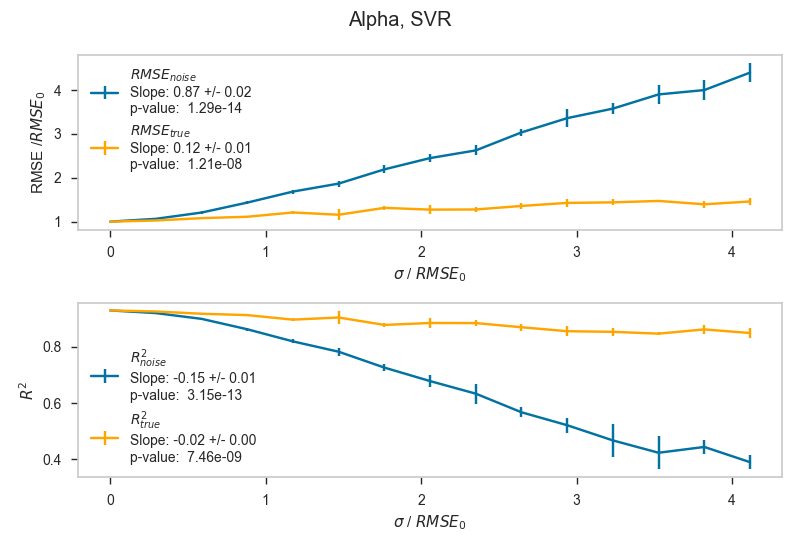

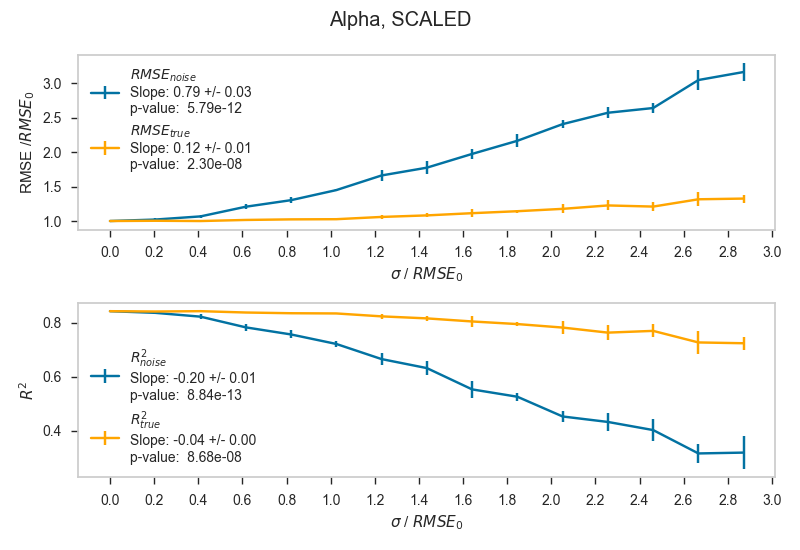


Ridge


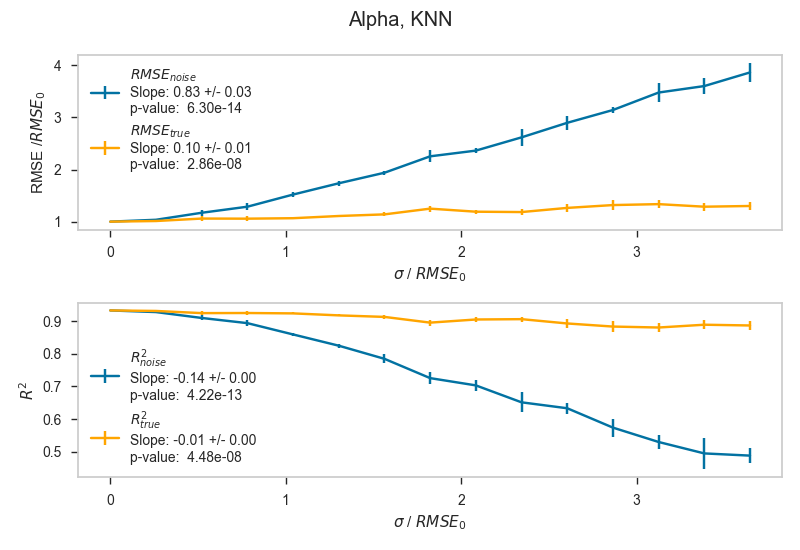


**Figure S4.** Plots showing RMSE, RMSE_true_, R^2^, and R^2^_true_ as functions of the standard deviation (σ) of the Gaussian distribution of the added random error for the Alpha dataset. Each plot is a separate algorithm. Blue lines represent metrics for predictions which are evaluated against Test_noise_, and orange lines represent metrics for predictions which are evaluated against Test_true_.


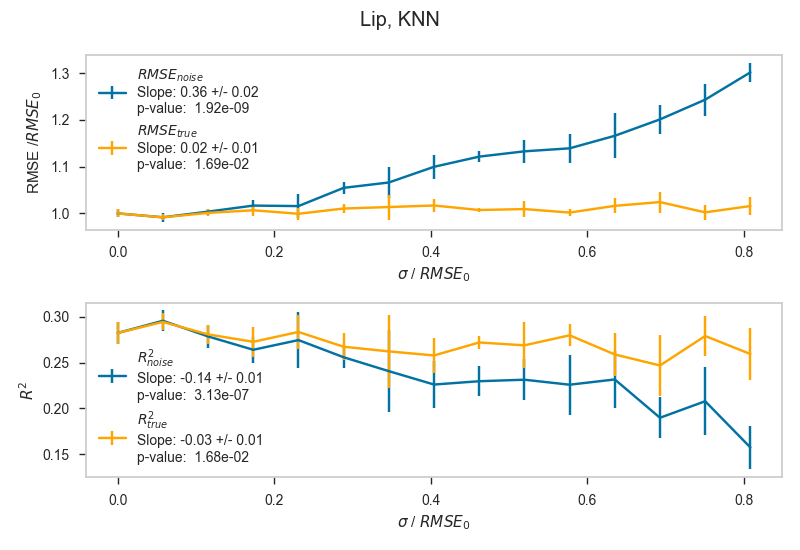

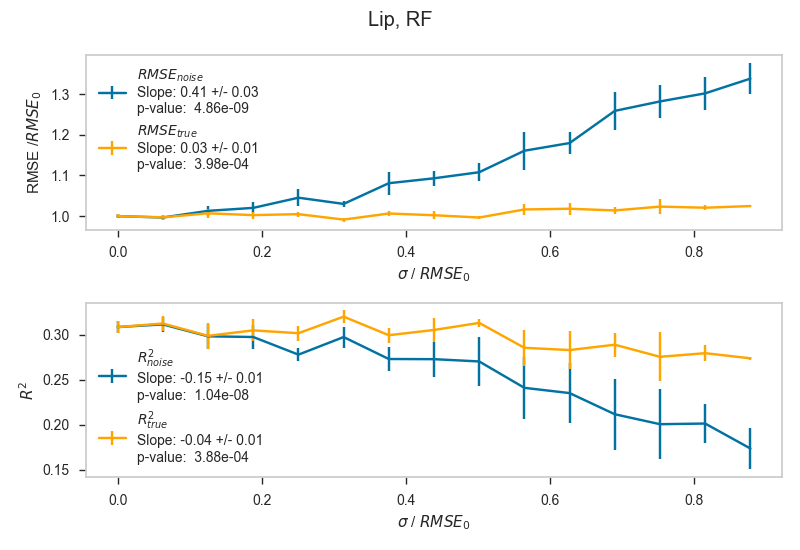

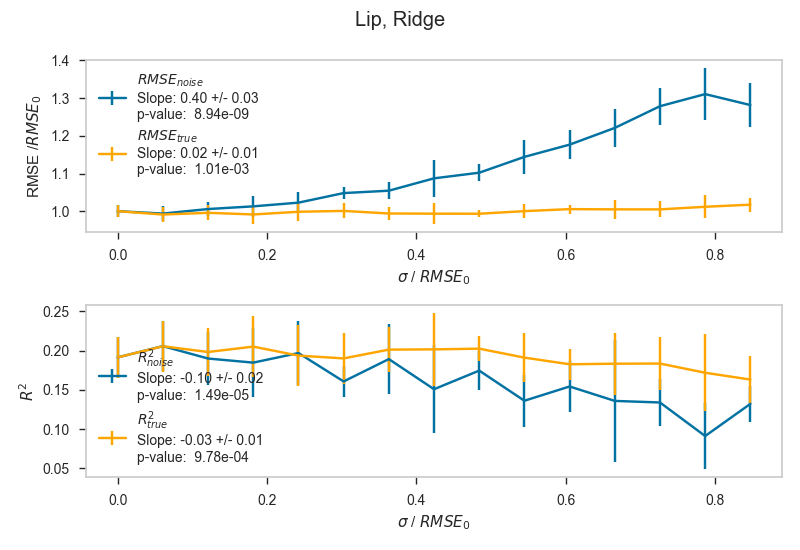

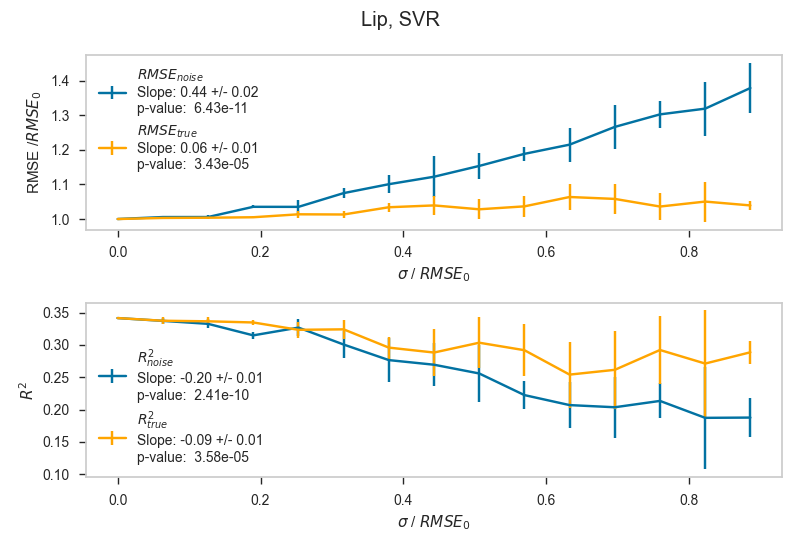


**Figure S5.** Plots showing RMSE, RMSE_true_, R^2^, and R^2^_true_ as functions of the standard deviation (σ) of the Gaussian distribution of the added random error for the Lip dataset. Each plot is a separate algorithm. Blue lines represent metrics for predictions which are evaluated against Test_noise_, and orange lines represent metrics for predictions which are evaluated against Test_true_.


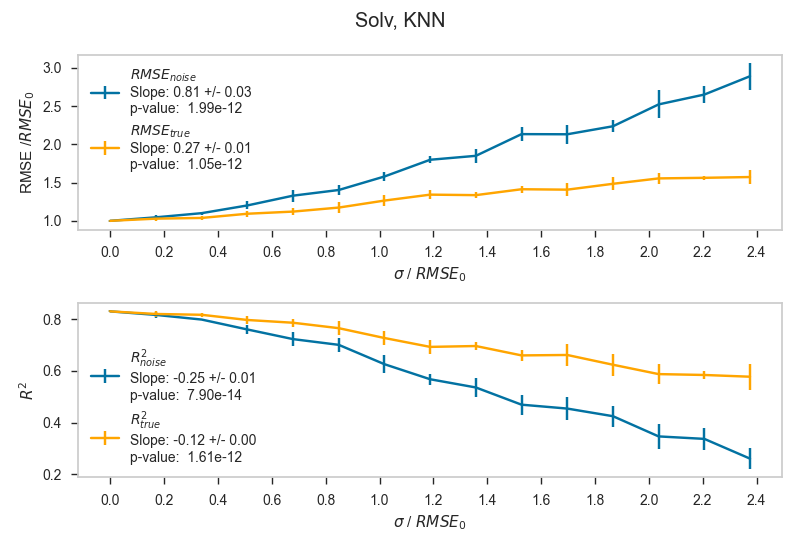

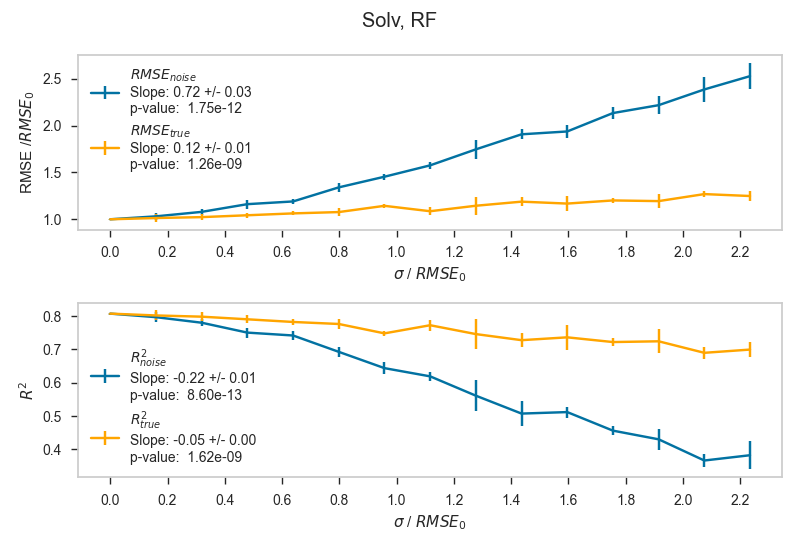

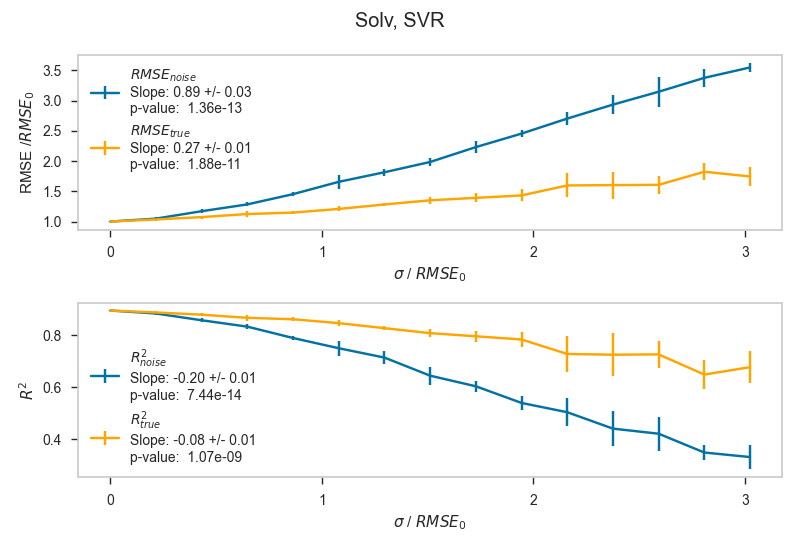

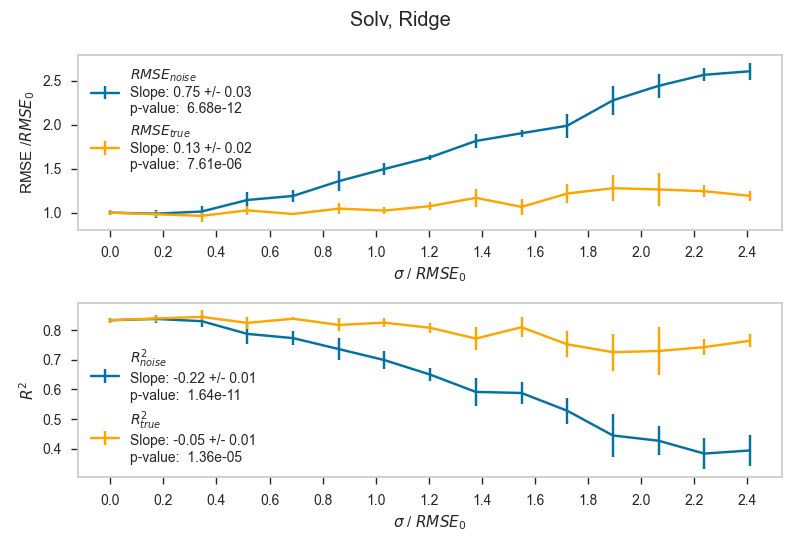


**Figure S6.** Plots showing RMSE, RMSE_true_, R^2^, and R^2^_true_ as functions of the standard deviation (σ) of the Gaussian distribution of the added random error for the Solv dataset. Each plot is a separate algorithm. Blue lines represent metrics for predictions which are evaluated against Test_noise_, and orange lines represent metrics for predictions which are evaluated against Test_true_.


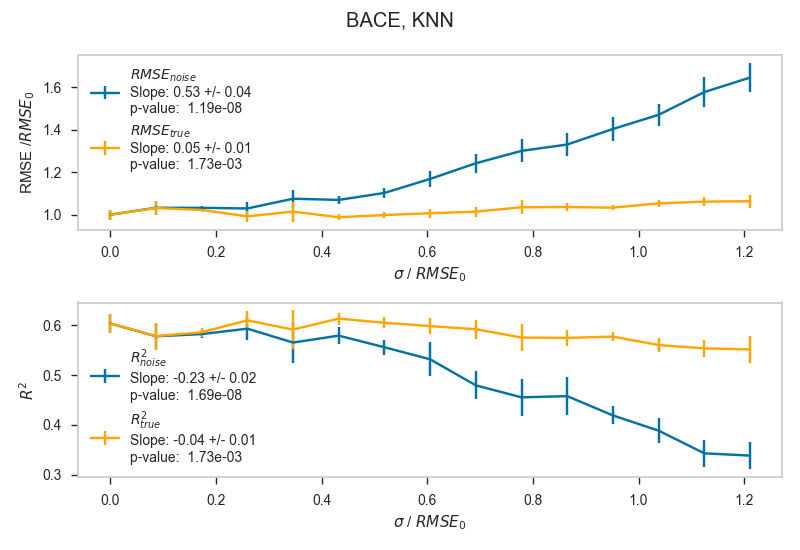

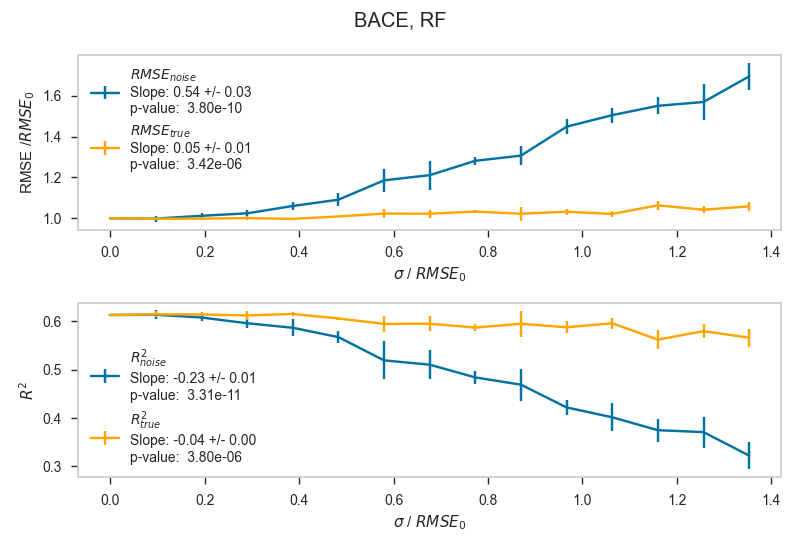

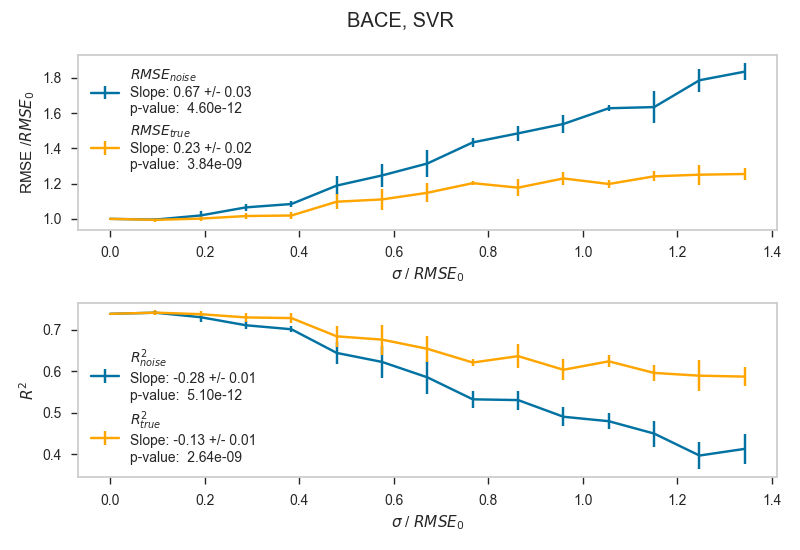

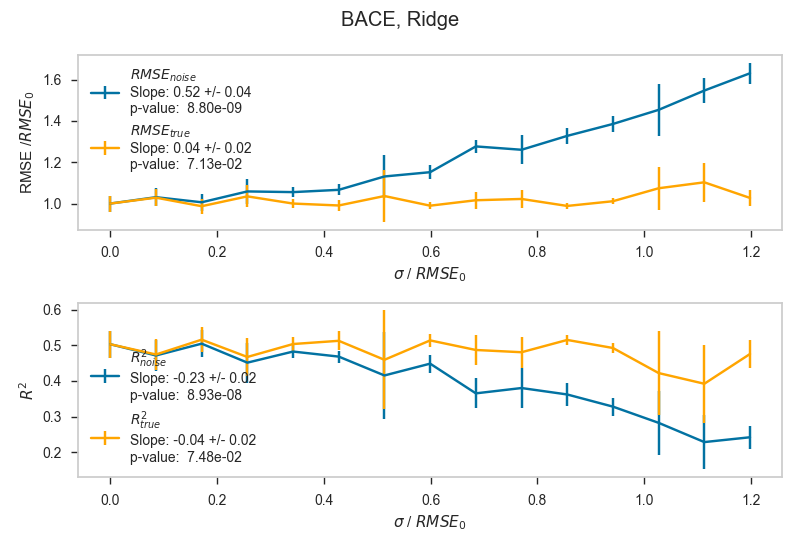


**Figure S7.** Plots showing RMSE, RMSE_true_, R^2^, and R^2^_true_ as functions of the standard deviation (σ) of the Gaussian distribution of the added random error for the BACE dataset. Each plot is a separate algorithm. Blue lines represent metrics for predictions which are evaluated against Test_noise_, and orange lines represent metrics for predictions which are evaluated against Test_true_.


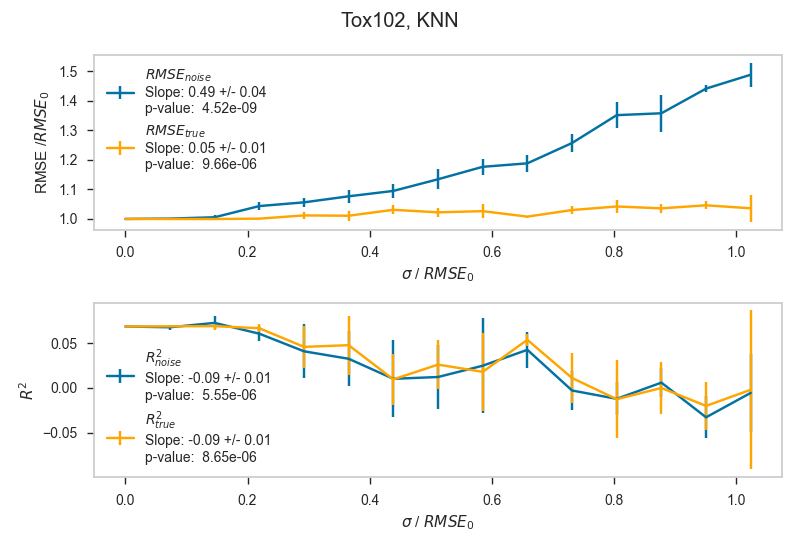

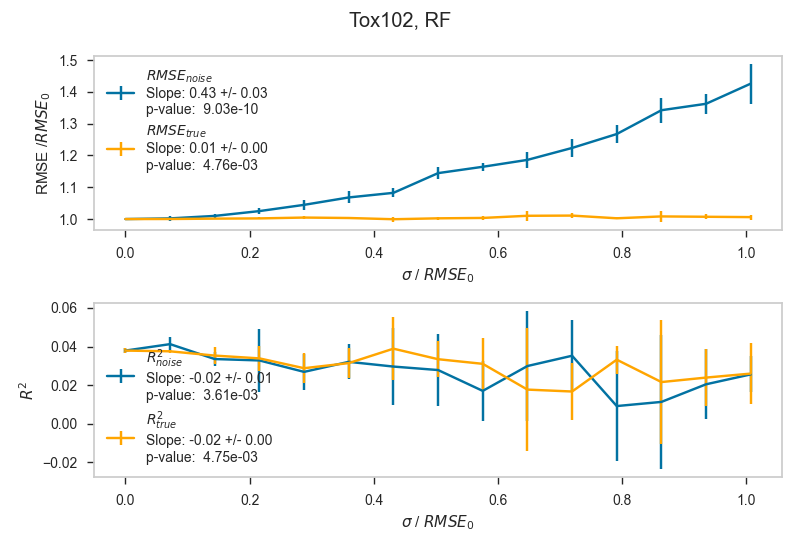

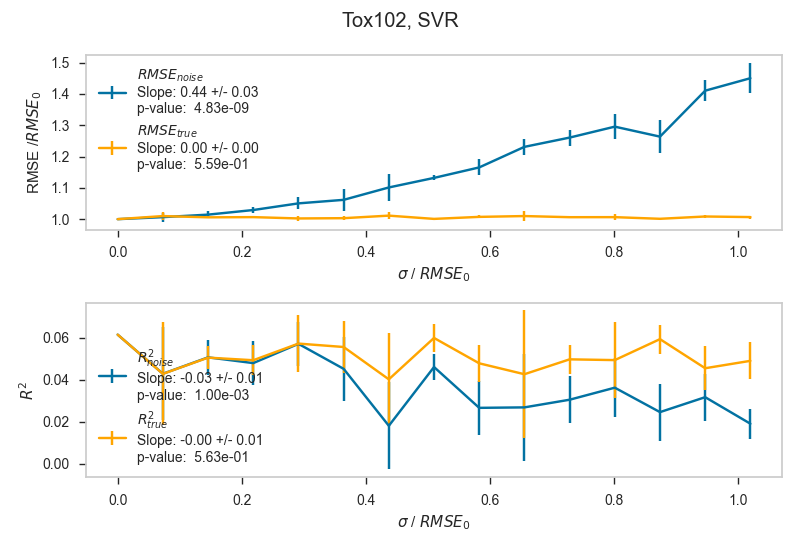

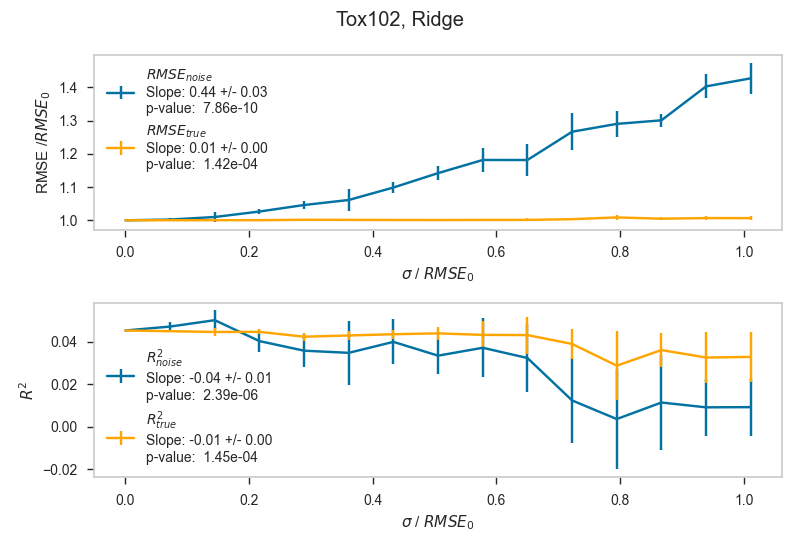


**Figure S8.** Plots showing RMSE, RMSE_true_, R^2^, and R^2^_true_ as functions of the standard deviation (σ) of the Gaussian distribution of the added random error for the Tox102 dataset. Each plot is a separate algorithm. Blue lines represent metrics for predictions which are evaluated against Test_noise_, and orange lines represent metrics for predictions which are evaluated against Test_true_.


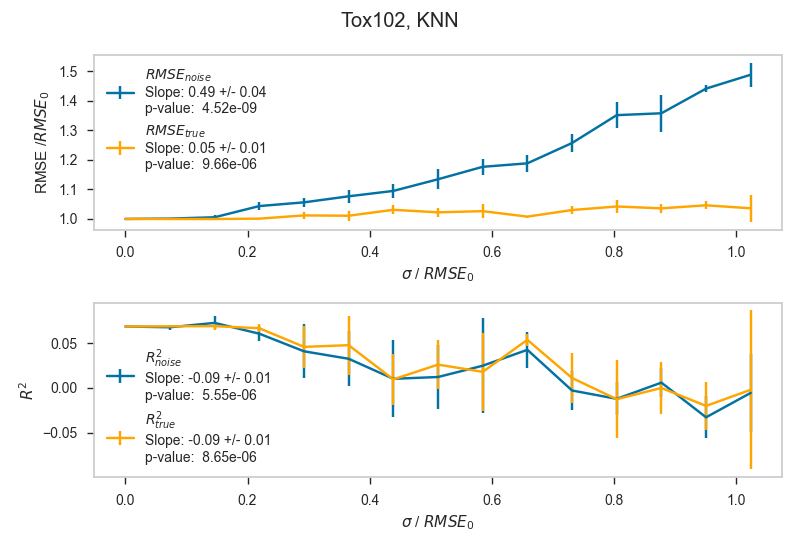

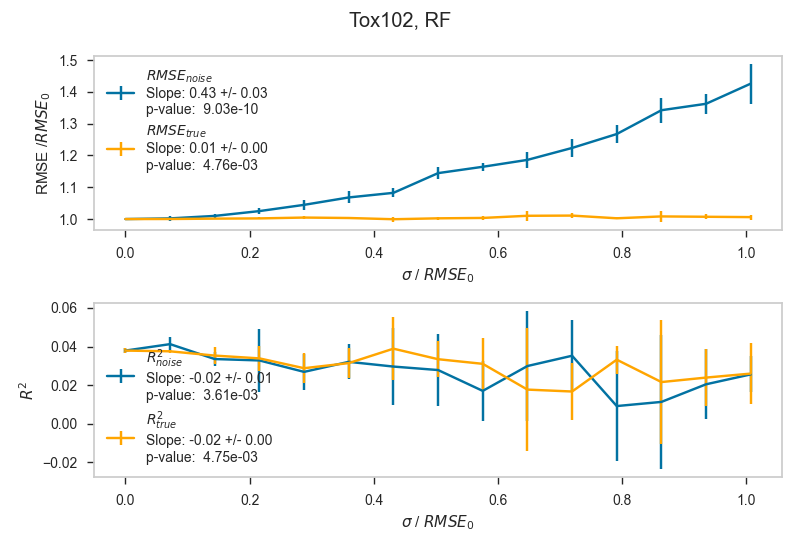

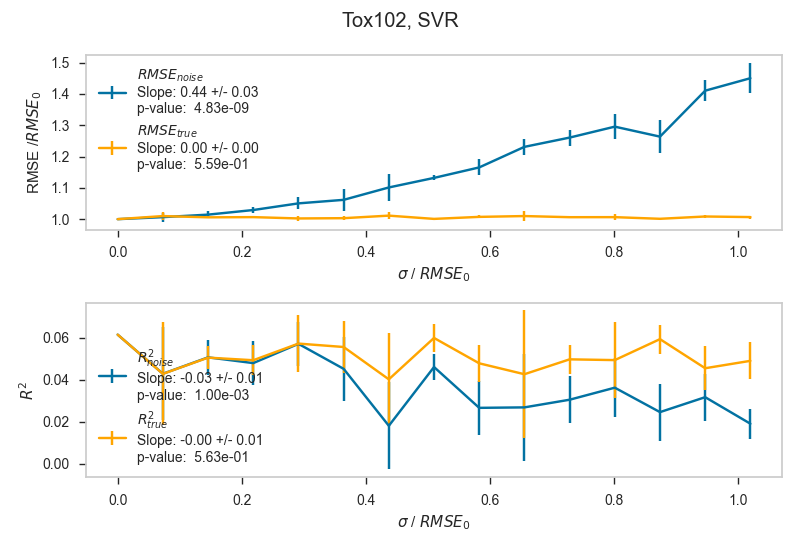

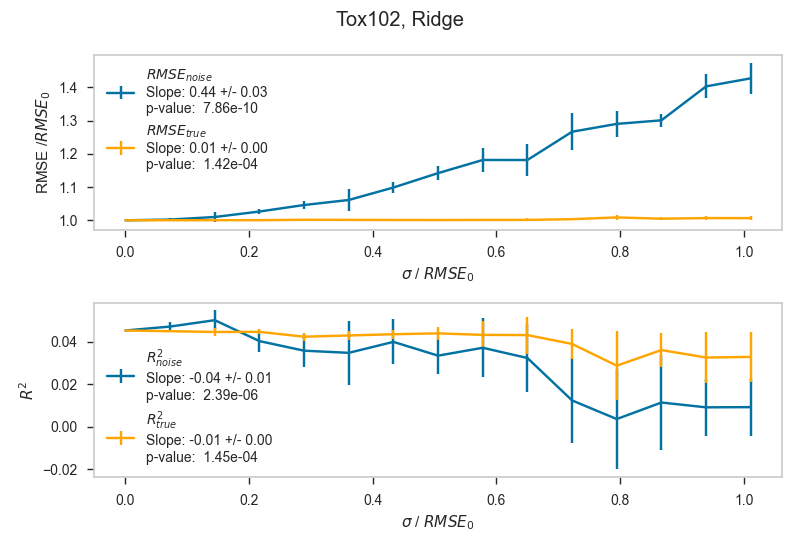


**Figure S9.** Plots showing RMSE, RMSE_true_, R^2^, and R^2^_true_ as functions of the standard deviation (σ) of the Gaussian distribution of the added random error for the Tox134 dataset. Each plot is a separate algorithm. Blue lines represent metrics for predictions which are evaluated against Test_noise_, and orange lines represent metrics for predictions which are evaluated against Test_true_.


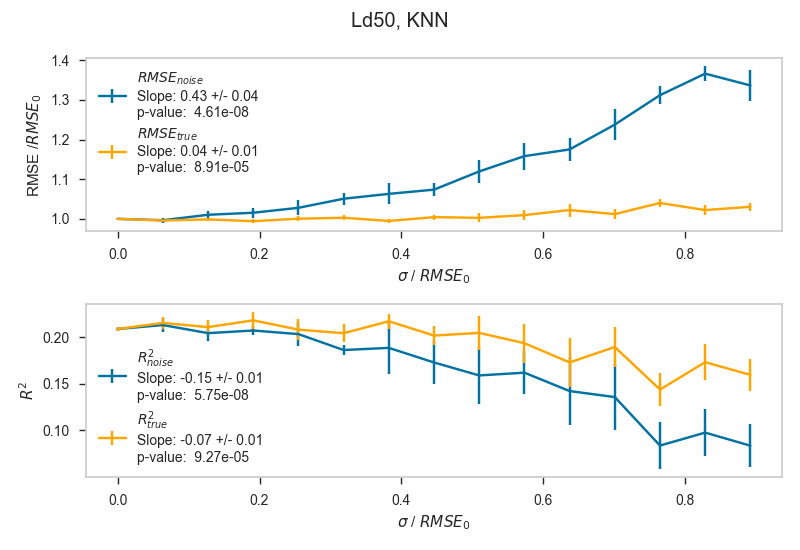

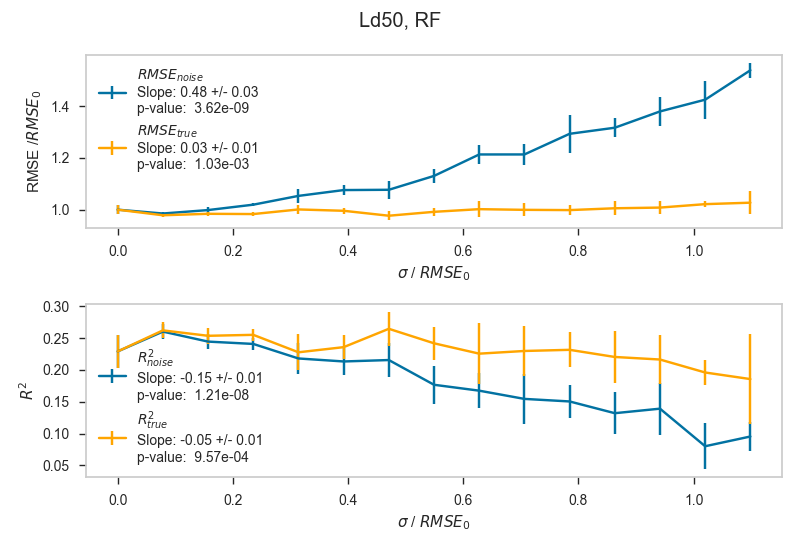

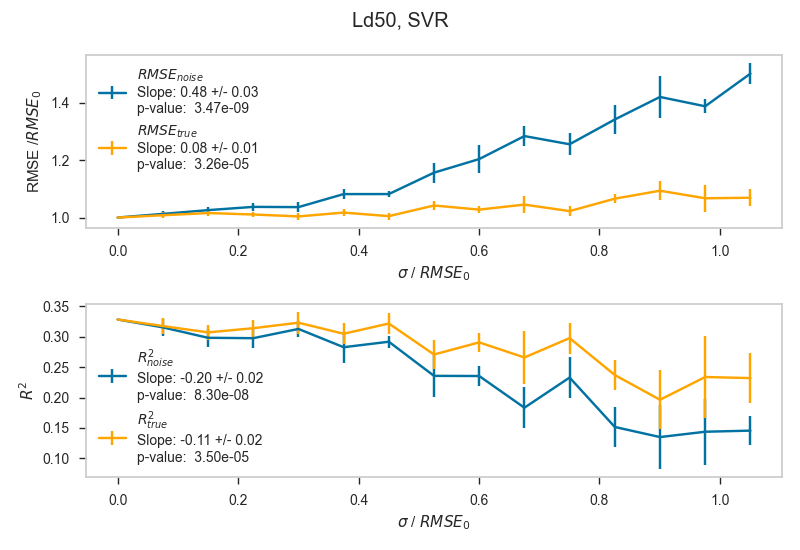

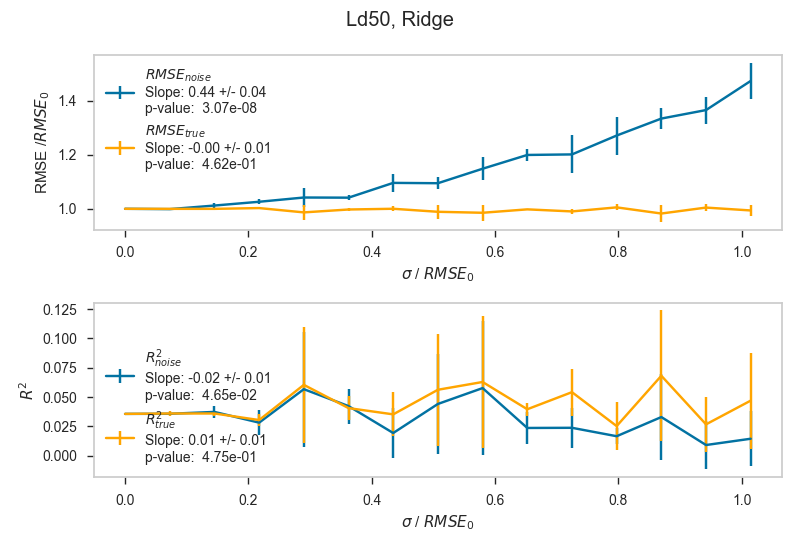


**Figure S10.** Plots showing RMSE, RMSE_true_, R^2^, and R^2^_true_ as functions of the standard deviation (σ) of the Gaussian distribution of the added random error for the logLD_50_ dataset. Each plot is a separate algorithm. Blue lines represent metrics for predictions which are evaluated against Test_noise_, and orange lines represent metrics for predictions which are evaluated against Test_true_.

## S3.2 Gaussian Process RMSE and R^2^ Plots


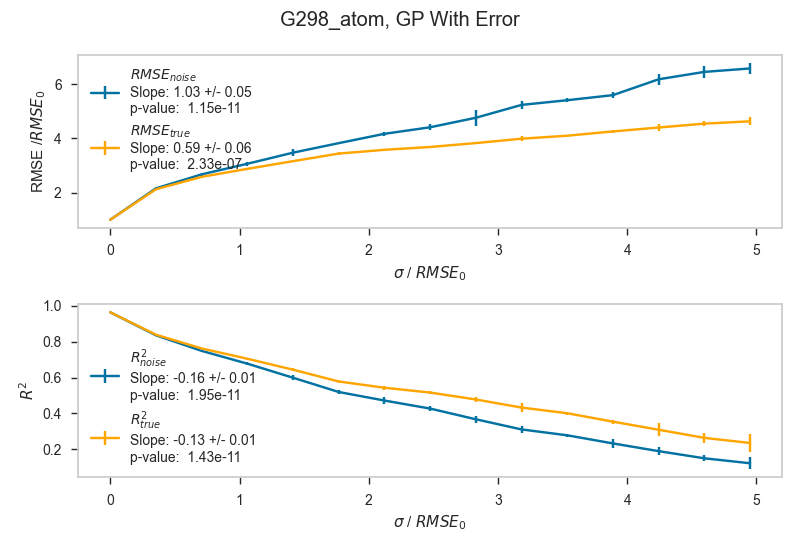

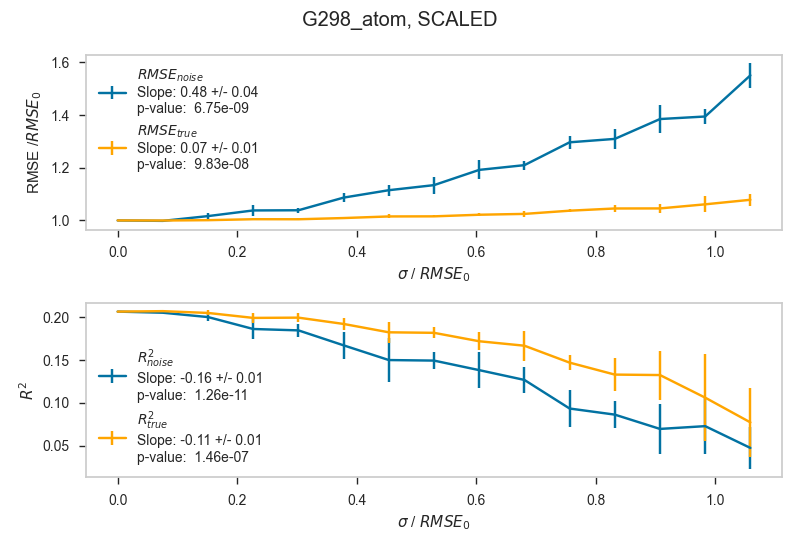


GP

**Figure S11.** Plots showing RMSE, RMSE_true_, R^2^, and R^2^_true_ as functions of the standard deviation (σ) of the Gaussian distribution of the added random error for the G298_atom dataset and the Gaussian Process algorithm. The left plot is for data where *no uncertainty information has been given* to the GP algorithm a priori, and the right plot is for data where uncertainty *has been given* to the GP algorithm. Blue lines represent metrics for predictions which are evaluated against Test_noise_, and orange lines represent metrics for predictions which are evaluated against Test_true_.


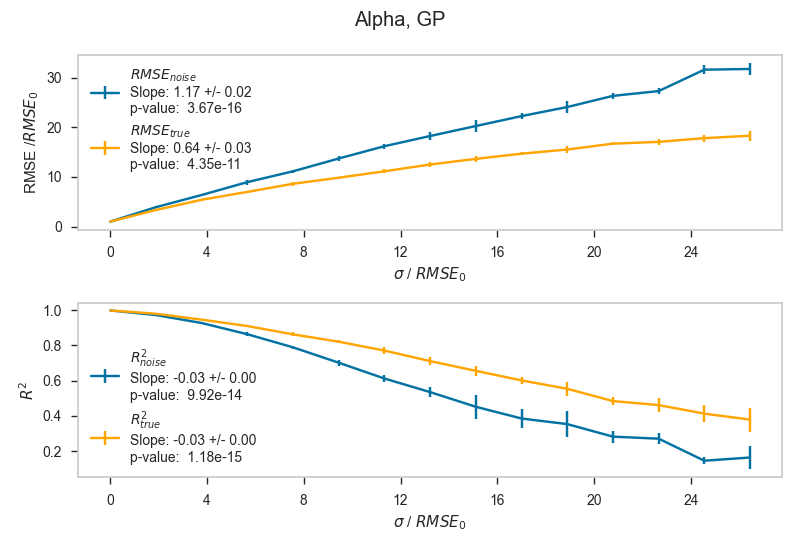

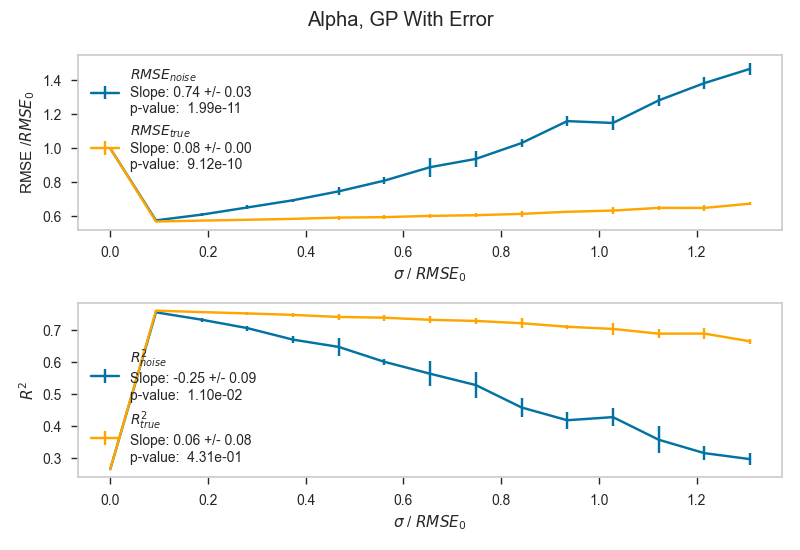


**Figure S12.** Plots showing RMSE, RMSE_true_, R^2^, and R^2^_true_ as functions of the standard deviation (σ) of the Gaussian distribution of the added random error for the Alpha dataset and the Gaussian Process algorithm. The left plot is for data where *no uncertainty information has been given* to the GP algorithm *a priori,* and the right plot is for data where uncertainty *has been given* to the GP algorithm. Blue lines represent metrics for predictions which are evaluated against Test_noise_, and orange lines represent metrics for predictions which are evaluated against Test_true_.


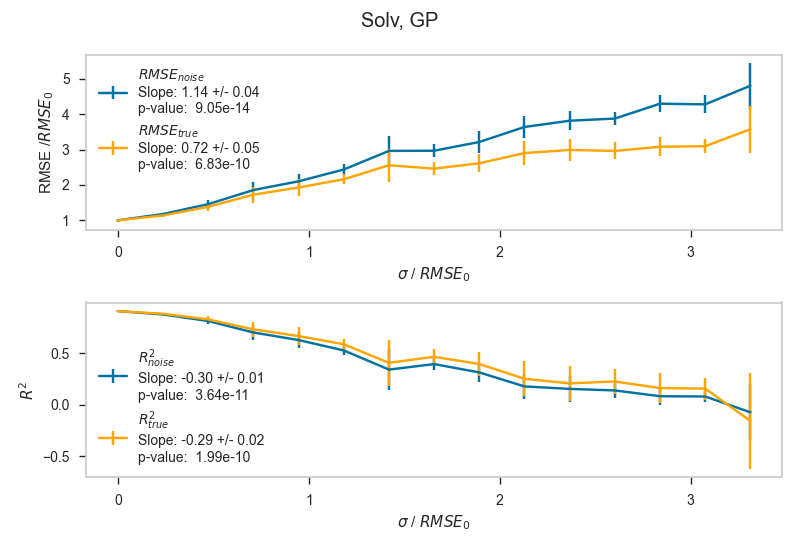

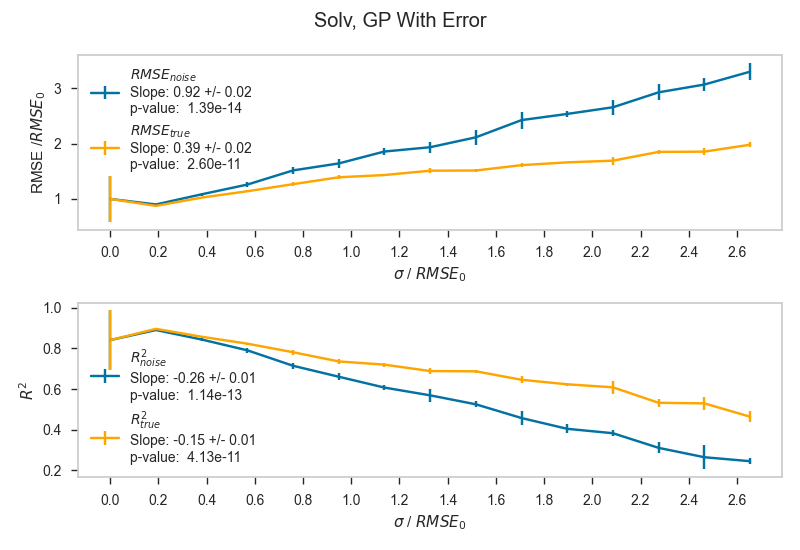


**Figure S13.** Plots showing RMSE, RMSE_true_, R^2^, and R^2^_true_ as functions of the standard deviation (σ) of the Gaussian distribution of the added random error for the Solv dataset and the Gaussian Process algorithm. The left plot is for data where *no uncertainty information has been given* to the GP algorithm *a priori,* and the right plot is for data where uncertainty *has been given* to the GP algorithm. Blue lines represent metrics for predictions which are evaluated against Test_noise_, and orange lines represent metrics for predictions which are evaluated against Test_true_.


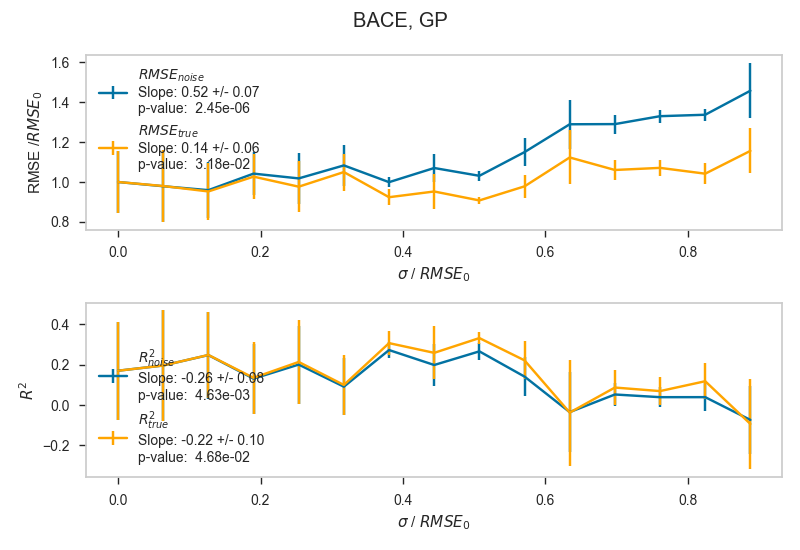

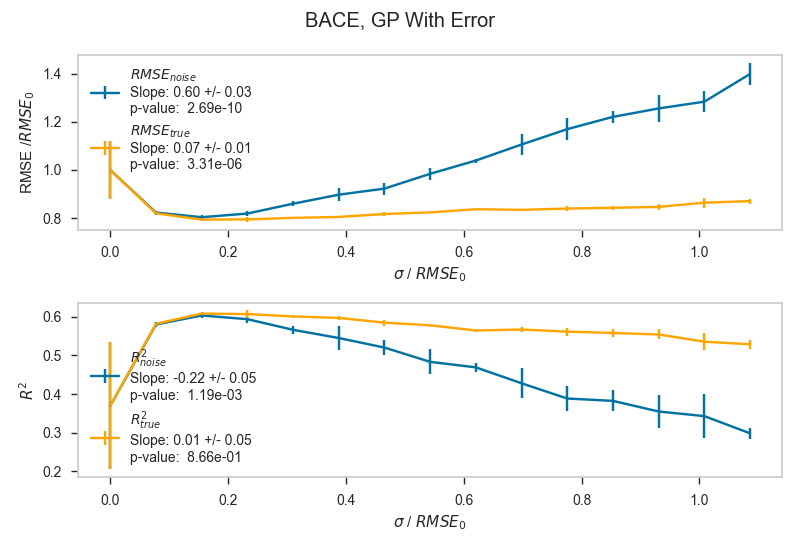


**Figure S14.** Plots showing RMSE, RMSE_true_, R^2^, and R^2^_true_ as functions of the standard deviation (σ) of the Gaussian distribution of the added random error for the BACE dataset and the Gaussian Process algorithm. The left plot is for data where *no uncertainty information has been given* to the GP algorithm *a priori,* and the right plot is for data where uncertainty *has been given* to the GP algorithm. Blue lines represent metrics for predictions which are evaluated against Test_noise_, and orange lines represent metrics for predictions which are evaluated against Test_true_.


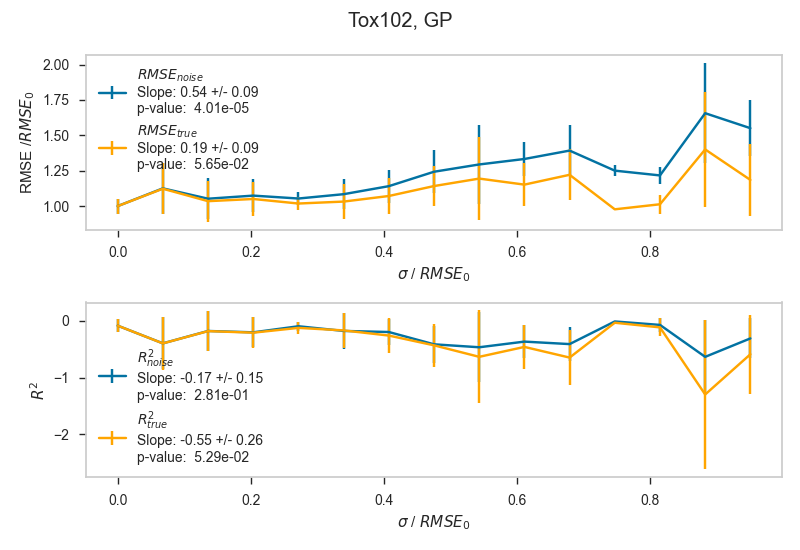

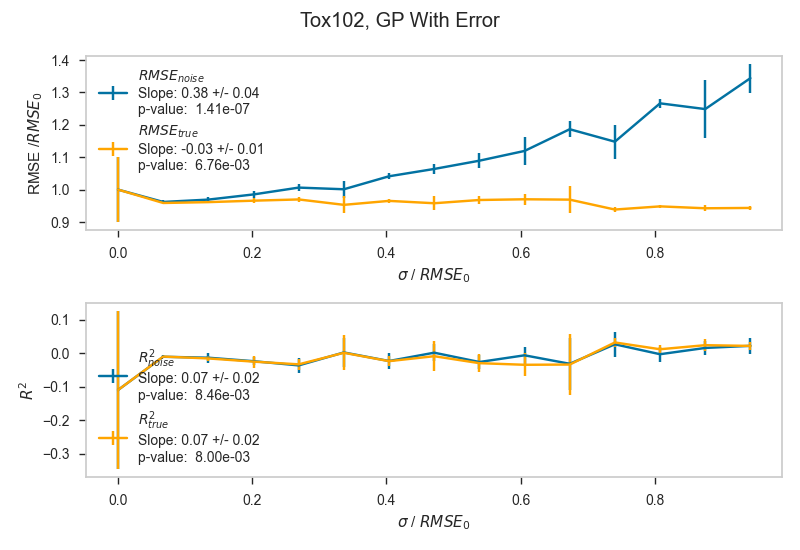


**Figure S15.** Plots showing RMSE, RMSE_true_, R^2^, and R^2^_true_ as functions of the standard deviation (σ) of the Gaussian distribution of the added random error for the Tox102 dataset and the Gaussian Process algorithm. The left plot is for data where *no uncertainty information has been given* to the GP algorithm *a priori,* and the right plot is for data where uncertainty *has been given* to the GP algorithm. Blue lines represent metrics for predictions which are evaluated against Test_noise_, and orange lines represent metrics for predictions which are evaluated against Test_true_.


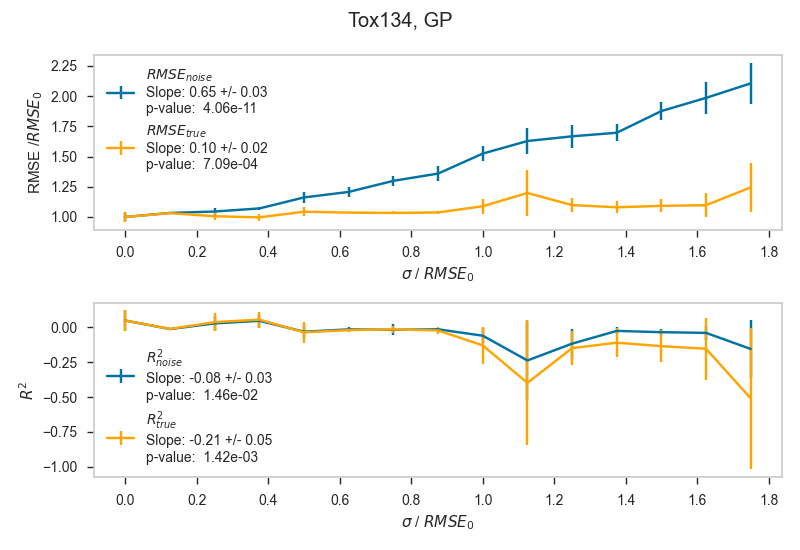

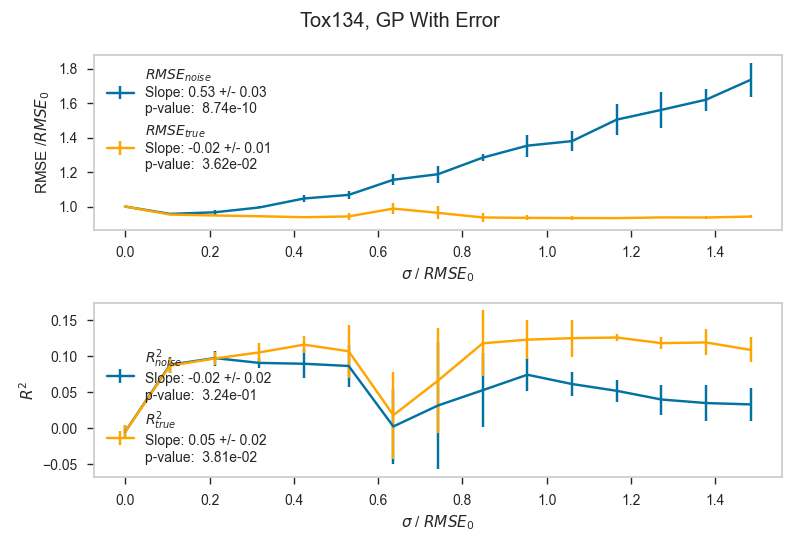


**Figure S16.** Plots showing RMSE, RMSE_true_, R^2^, and R^2^_true_ as functions of the standard deviation (σ) of the Gaussian distribution of the added random error for the Tox134 dataset and the Gaussian Process algorithm. The left plot is for data where *no uncertainty information has been given* to the GP algorithm *a priori,* and the right plot is for data where uncertainty *has been given* to the GP algorithm. Blue lines represent metrics for predictions which are evaluated against Test_noise_, and orange lines represent metrics for predictions which are evaluated against Test_true_.


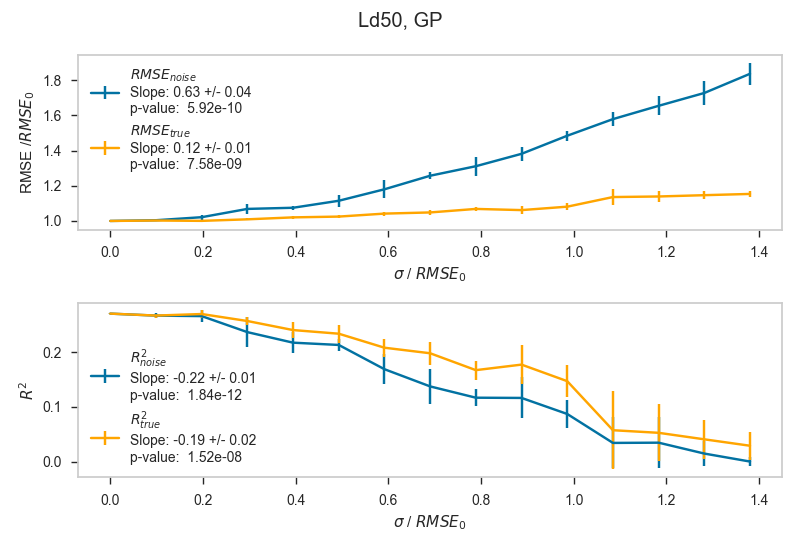

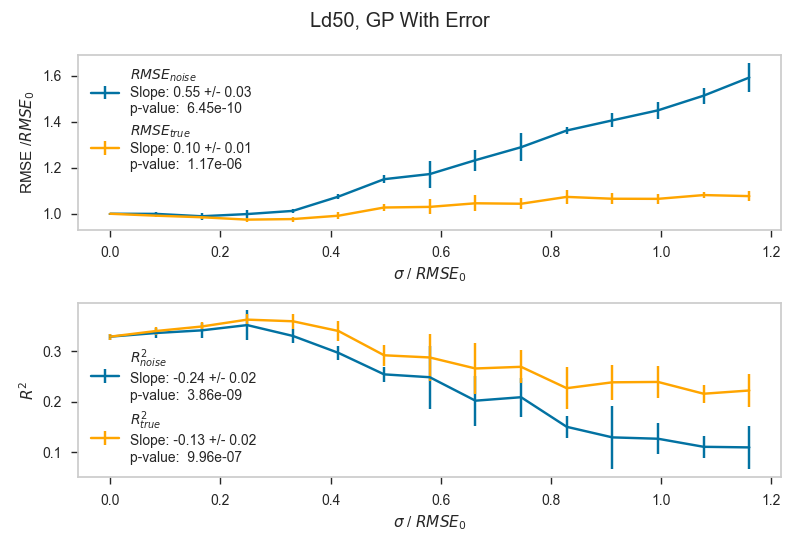


**Figure S17.** Plots showing RMSE, RMSE_true_, R^2^, and R^2^_true_ as functions of the standard deviation (σ) of the Gaussian distribution of the added random error for the LD_50_ dataset and the Gaussian Process algorithm. The left plot is for data where *no uncertainty information has been given* to the GP algorithm *a priori,* and the right plot is for data where uncertainty *has been given* to the GP algorithm. Blue lines represent metrics for predictions which are evaluated against Test_noise_, and orange lines represent metrics for predictions which are evaluated against Test_true_.

## S3.3 Gaussian Process Prediction Error Plots

**Figure S18.** Plots of mean prediction error (σ_ŷ_) and prediction error 95% Confidence Interval as functions of the standard deviation (σ) of the Gaussian distribution of the added random error for the G298_atom dataset. The left plot is for data where *no uncertainty information has been given* to the GP algorithm *a priori,* and the right plot is for data where uncertainty *has been given* to the GP algorithm.


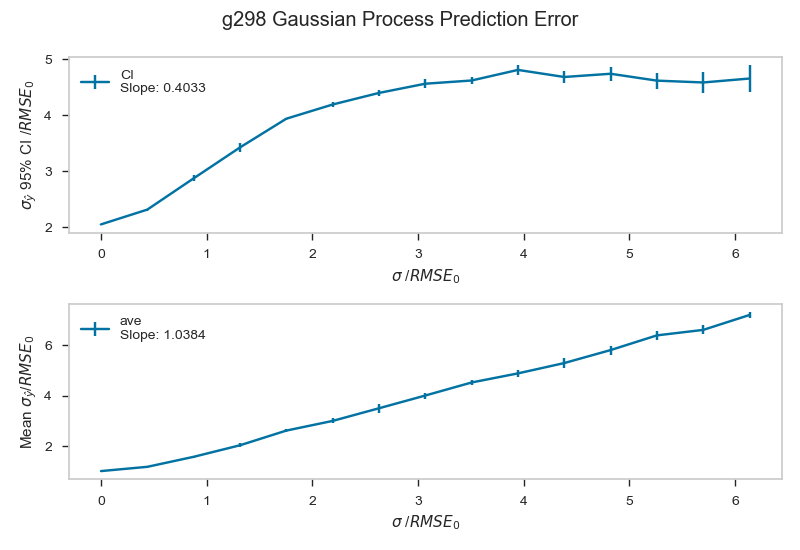

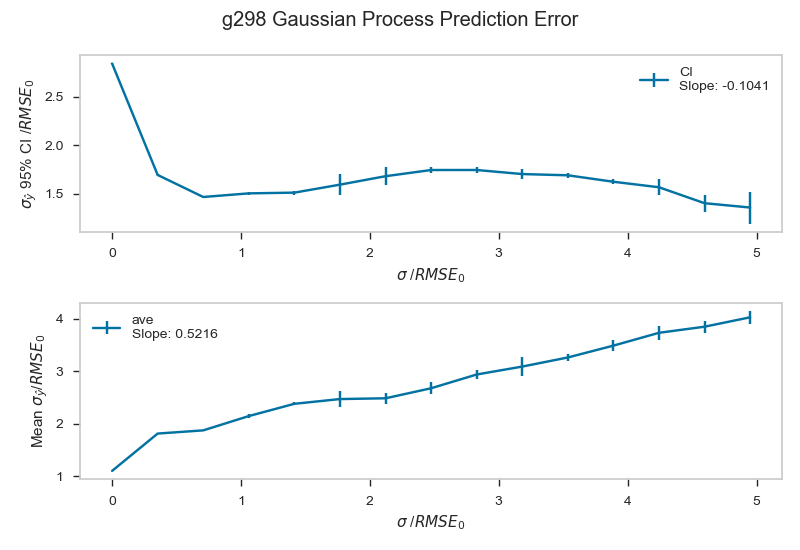


**Figure S19.** Plots of mean prediction error (σ_ŷ_) and prediction error 95% Confidence Interval as functions of the standard deviation (σ) of the Gaussian distribution of the added random error for the Alpha dataset. The left plot is for data where *no uncertainty information has been given* to the GP algorithm *a priori,* and the right plot is for data where uncertainty *has been given* to the GP algorithm.


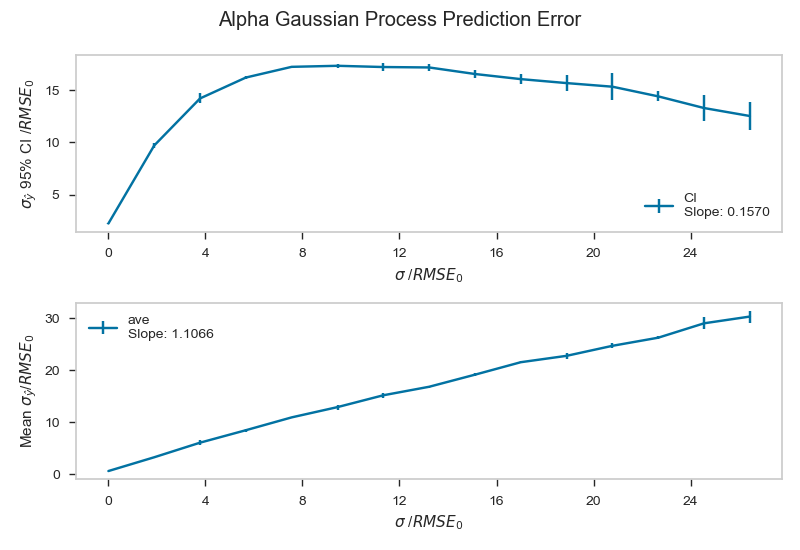

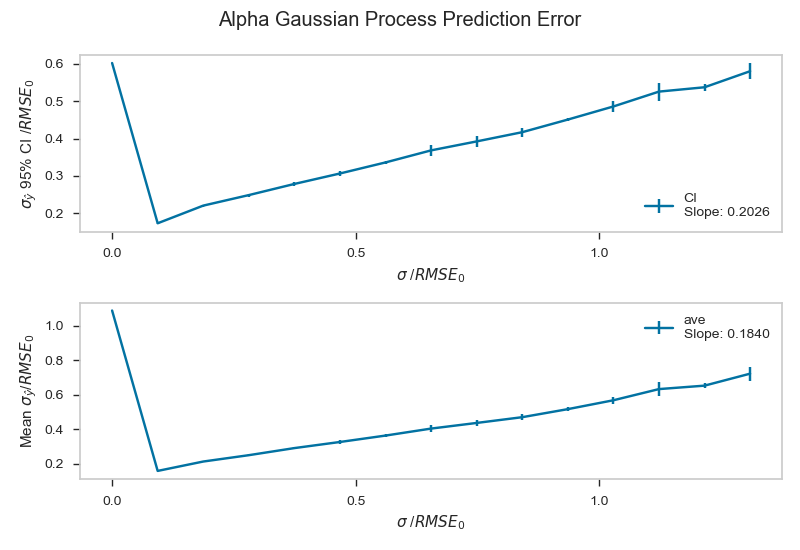


**Figure S20.** Plots of mean prediction error (σ_ŷ_) and prediction error 95% Confidence Interval as functions of the standard deviation (σ) of the Gaussian distribution of the added random error for the Solv dataset. The left plot is for data where *no uncertainty information has been given* to the GP algorithm *a priori,* and the right plot is for data where uncertainty *has been given* to the GP algorithm.


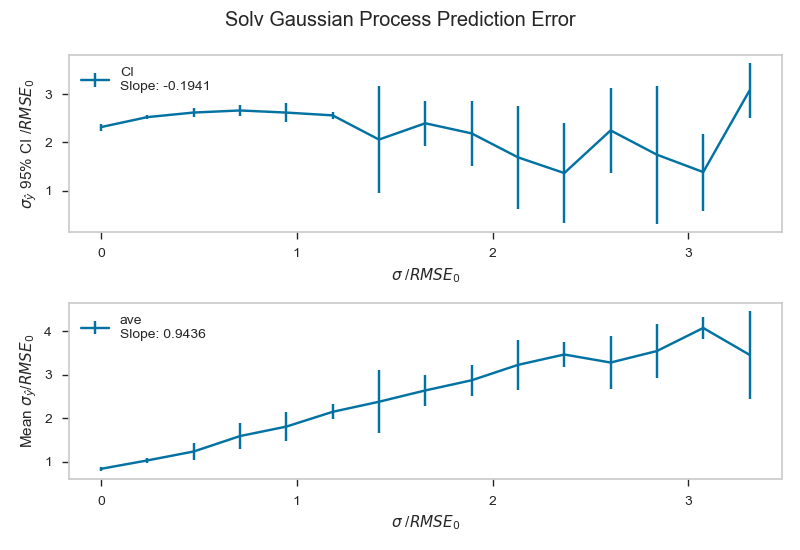

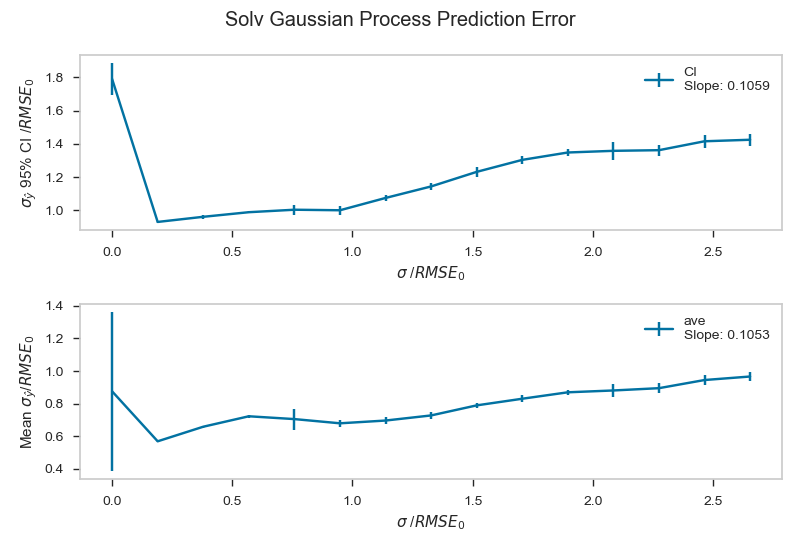


**Figure S21.** Plots of mean prediction error (σ_ŷ_) and prediction error 95% Confidence Interval as functions of the standard deviation (σ) of the Gaussian distribution of the added random error for the BACE dataset. The left plot is for data where *no uncertainty information has been given* to the GP algorithm *a priori,* and the right plot is for data where uncertainty *has been given* to the GP algorithm.


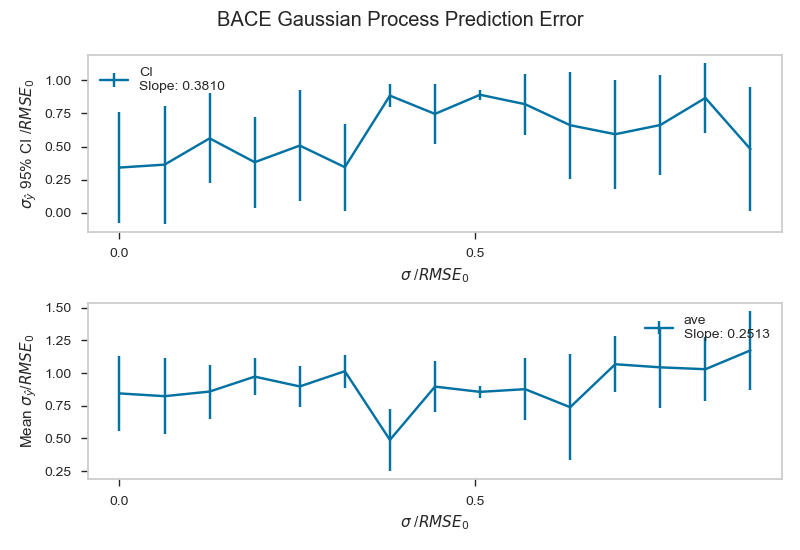

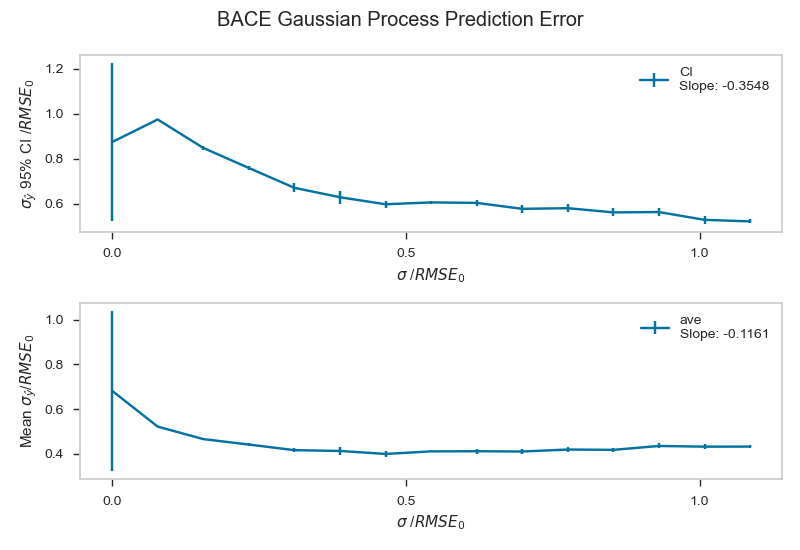


**Figure S22.** Plots of mean prediction error (σ_ŷ_) and prediction error 95% Confidence Interval as functions of the standard deviation (σ) of the Gaussian distribution of the added random error for the Tox102 dataset. The left plot is for data where *no uncertainty information has been given* to the GP algorithm *a priori,* and the right plot is for data where uncertainty *has been given* to the GP algorithm.


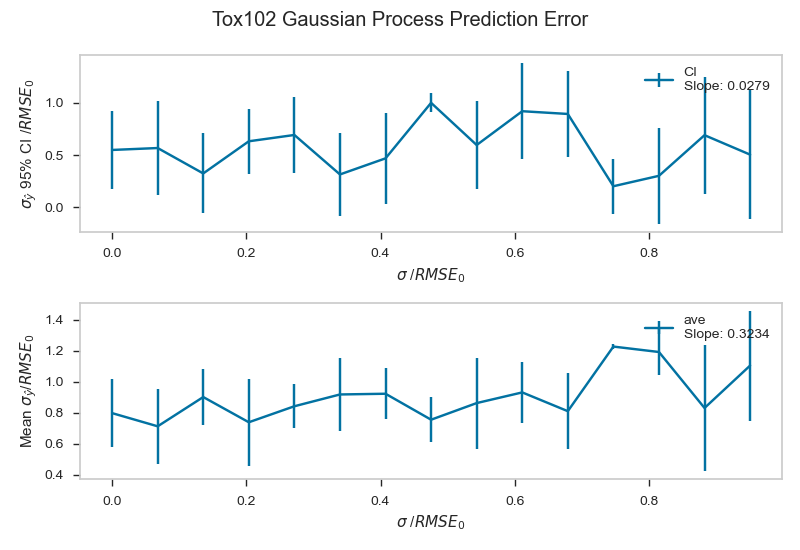

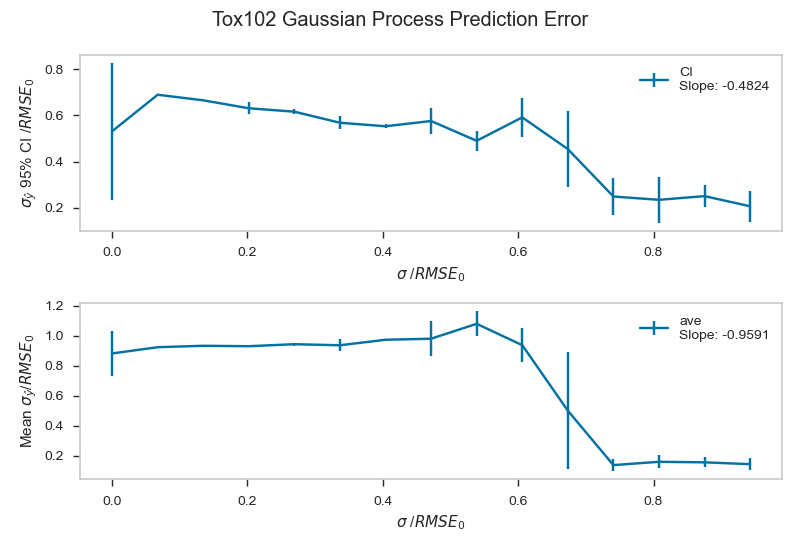


**Figure S23.** Plots of mean prediction error (σ_ŷ_) and prediction error 95% Confidence Interval as functions of the standard deviation (σ) of the Gaussian distribution of the added random error for the Tox134 dataset. The left plot is for data where *no uncertainty information has been given* to the GP algorithm *a priori,* and the right plot is for data where uncertainty *has been given* to the GP algorithm.


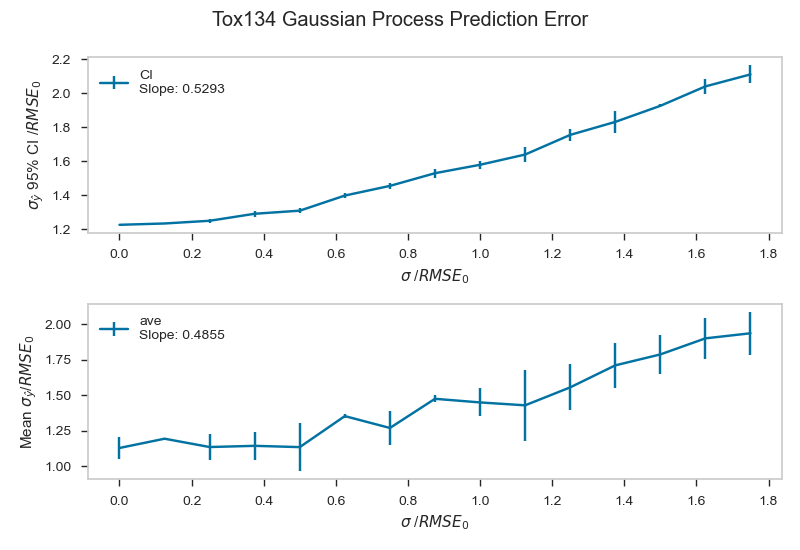

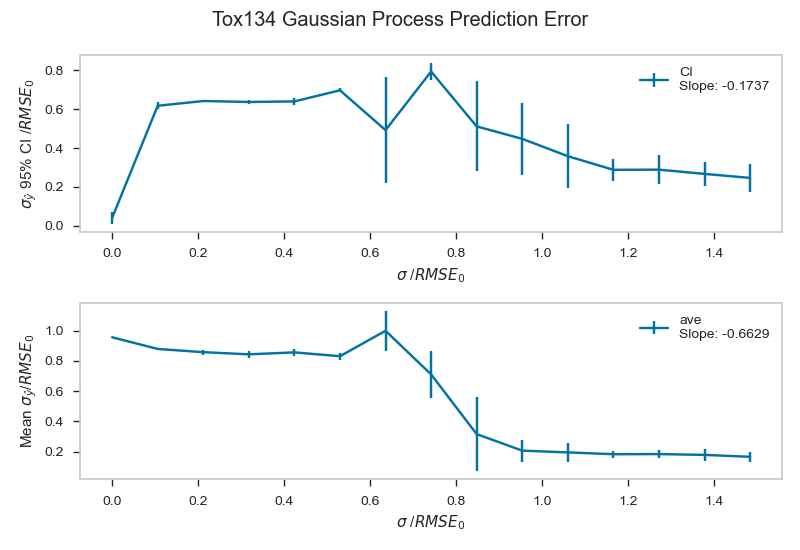


**Figure S24.** Plots of mean prediction error (σ_ŷ_) and prediction error 95% Confidence Interval as functions of the standard deviation (σ) of the Gaussian distribution of the added random error for the LD_50_ dataset. The left plot is for data where *no uncertainty information has been given* to the GP algorithm *a priori,* and the right plot is for data where uncertainty *has been given* to the GP algorithm.


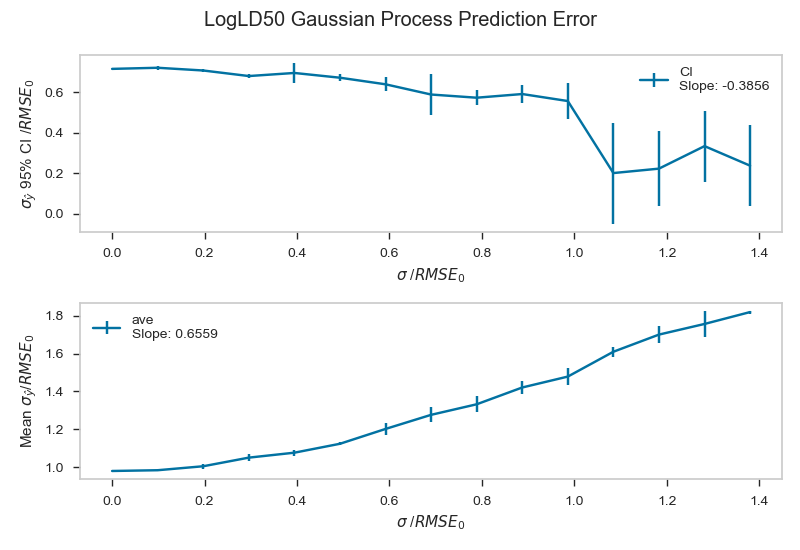

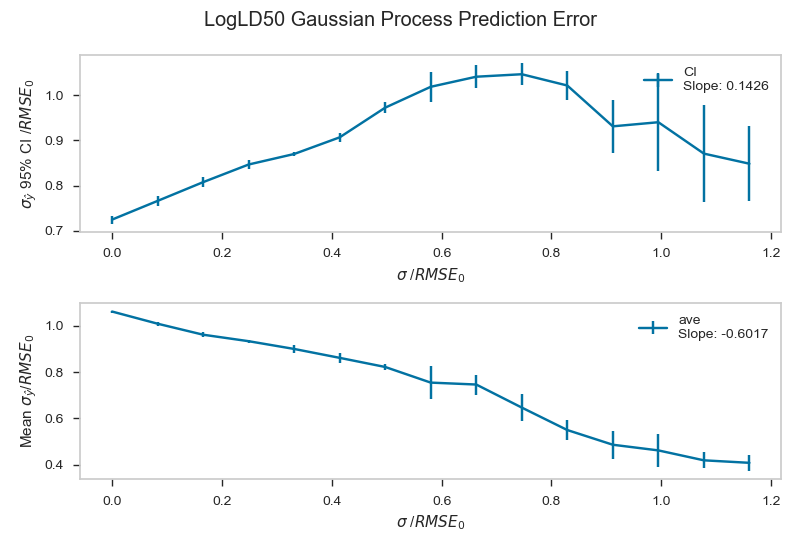


# S4. Tables

## S4.1 Sigma Values

The vector Y_noise_ was created by adding a unique term sampled from a Gaussian distribution to each term in Y according to the following equation:

$$Y_{{noise}_{n},i}=Y+N(0, \sigma_{{noise}_{n}})$$

Sigma values were generated using the following formula:

$$\sigma_{{noise}_{n}}={(Y}_{max}-Y_{min})*n*multiplier$$

**Table S1.** Sigma values for each dataset.

| *n*/ Dataset | **G298atom** | **Alpha** | **Lip** | **Solv** | **BACE** | **Tox102** | **Tox134** | **LD50** |
| --- | --- | --- | --- | --- | --- | --- | --- | --- |
| **1** | 0.00 | 0.00 | 0.00 | 0.00 | 0.00 | 0.00 | 0.00 | 0.00 |
| **2** | 16.8 | 0.195 | 0.0601 | 0.289 | 0.0781 | 0.0472 | 0.0634 | 0.0645 |
| **3** | 33.6 | 0.391 | 0.120 | 0.578 | 0.156 | 0.0943 | 0.126 | 0.127 |
| **4** | 50.4 | 0.586 | 0.179 | 0.867 | 0.235 | 0.140 | 0.189 | 0.191 |
| **5** | 67.2 | 0.782 | 0.239 | 1.16 | 0.313 | 0.187 | 0.253 | 0.254 |
| **6** | 84.0 | 0.977 | 0.299 | 1.45 | 0.391 | 0.234 | 0.316 | 0.318 |
| **7** | 100 | 1.17 | 0.359 | 1.73 | 0.469 | 0.281 | 0.279 | 0.381 |
| **8** | 118 | 1.37 | 0.419 | 2.02 | 0.548 | 0.328 | 0.442 | 0.445 |
| **9** | 134 | 1.56 | 0.478 | 2.31 | 0.626 | 0.374 | 0.505 | 0.508 |
| **10** | 151 | 1.76 | 0.538 | 2.60 | 0.704 | 0.421 | 0.568 | 0.572 |
| **11** | 168 | 1.95 | 0.598 | 2.89 | 0.782 | 0.468 | 0.631 | 0.635 |
| **12** | 185 | 2.15 | 0.658 | 3.18 | 0.861 | 0.515 | 0.694 | 0.699 |
| **13** | 202 | 2.34 | 0.718 | 3.47 | 0.939 | 0.562 | 0.758 | 0.762 |
| **14** | 218 | 2.54 | 0.777 | 3.76 | 1.02 | 0.609 | 0.821 | 0.826 |
| **15** | 235 | 2.74 | 0.837 | 4.05 | 1.10 | 0.655 | 0.884 | 0.889 |

## S4.2 Results without Principal Component Analysis

To examine the effect of using principal component analysis (PCA) in the workflow, results were generated without PCA in the machine learning pipeline. This experiment was not performed with random forest (RF) because computational time scales with number of descriptors, making the computational time unreasonable. Each slope was determined to be statistically significant with *p*-values several orders of magnitude below 0.05.

**Table S2.** Slopes *m* and *m_true_* for each dataset and algorithm, without PCA.

| Dataset | Slope | Ridge | kNN | SVR |
| --- | --- | --- | --- | --- |
| G298_atom | *m_noise_* | 1.3 ± 0.040 | 0.73 ± 0.032 | 0.77 ± 0.034 |
|  | *m_true_* | 0.92 ± 0.041 | 0.091 ± 0.013 | 0.15 ± 0.00 |
| Alpha | *m_noise_* | 1.1 ± 0.032 | 0.66 ± 0.041 | 0.80 ± 0.030 |
|  | *m_true_* | 0.64 ± 0.031 | 0.05 ±0.010 | 0.17 ± 0.015 |
| Lip | *m_noise_* | 0.69 ± 0.072 | 0.36 ± 0.034 | 0.56 ± 0.030 |
|  | *m_true_* | 0.36 ± 0.064 | 0.03 ± 0.01 | 0.18 ± 0.011 |
| Solv | *m_noise_* | 1.3 ± 0.034 | 0.84 ± 0.032 | 0.77 ± 0.042 |
|  | *m_true_* | 0.95 ± 0.030 | 0.34 ± 0.020 | 0.23 ± 0.021 |
| BACE | *m_noise_* | 1.3 ± 0.033 | 0.89 ± 0.026 | 0.69 ± 0.032 |
|  | *m_true_* | 0.79 ± 0.032 | 0.13 ± 0.015 | 0.05 ± 0.00 |
| Tox_102 | *m_noise_* | 1.5 ± 0.030 | 0.89 ± 0.025 | 0.84 ± 0.032 |
|  | *m_true_* | 1.0 ± 0.030 | 0.14 ± 0.012 | 0.11 ± 0.00 |
| Tox_134 | *m_noise_* | 10.9 ± 0.820 | 0.86 ± 0.034 | 0.73 ± 0.032 |
|  | *m_true_* | 10.9 ± 0.820 | 0.11 ± 0.012 | 0.07 ± 0.00 |
| LD50 | *m_noise_* | 1.7 ± 0.080 | 0.82 ± 0.031 | 0.62 ± 0.035 |
|  | *m_true_* | 1.3 ± 0.090 | 0.11 ± 0.011 | 0.02 ± 0.00 |

**Table S3.** Ratios of *m_noise_/m_true_* for each dataset and algorithm without PCA.

| Dataset/Algorithm | Ridge | kNN | SVR |
| --- | --- | --- | --- |
| G_298_atom | 1.4 ± 0.10 | 8.0 ± 1.4 | 5.1 ± 0.22 |
| Alpha | 1.7 ± 0.13 | 13 ± 3.4 | 4.7 ± 0.58 |
| Lip | 1.9 ± 0.53 | 12 ± 5.0 | 3.1 ± 0.26 |
| Solv | 1.4 ± 0.080 | 2.5 ± 0.24 | 3.3 ± 0.48 |
| BACE | 1.6 ± 0.10 | 6.8 ± 0.95 | 14 ± 0.64 |
| Tox_102 | 1.5 ± 0.075 | 6.4 ± 0.70 | 7.6 ± 0.29 |
| Tox_134 | 1.0 ± 0.15 | 7.8 ± 1.2 | 10 ± 0.44 |
| LD50 | 1.3 ± 0.15 | 7.5 ± 1.0 | 31 ± 1.7 |

References

1. Wu, Z.; Ramsundar, B.; Feinberg, Evan N.; Gomes, J.; Geniesse, C.; Pappu, A. S.; Leswing, K.; Pande, V., MoleculeNet: a benchmark for molecular machine learning. *Chemical Science* **2018,** *9* (2), 513-530.

2. Blum, L. C.; Reymond, J.-L., 970 Million Druglike Small Molecules for Virtual Screening in the Chemical Universe Database GDB-13. *Journal of the American Chemical Society* **2009,** *131* (25), 8732-8733.

3. Ramakrishnan, R.; Dral, P. O.; Rupp, M.; von Lilienfeld, O. A., Quantum chemistry structures and properties of 134 kilo molecules. *Scientific Data* **2014,** *1* (1), 140022.

4. Wenlock, M.; Tomkinson, N. ChEMBL. <https://www.ebi.ac.uk/chembl/document_report_card/CHEMBL3301361/>.

5. Mobley, D. L.; Guthrie, J. P., FreeSolv: a database of experimental and calculated hydration free energies, with input files. *J Comput Aided Mol Des* **2014,** *28* (7), 711-720.

6. Subramanian, G.; Ramsundar, B.; Pande, V.; Denny, R. A., Computational Modeling of β-Secretase 1 (BACE-1) Inhibitors Using Ligand Based Approaches. *Journal of Chemical Information and Modeling* **2016,** *56* (10), 1936-1949.

7. Dix, D. J.; Houck, K. A.; Martin, M. T.; Richard, A. M.; Setzer, R. W.; Kavlock, R. J., The ToxCast Program for Prioritizing Toxicity Testing of Environmental Chemicals. *Toxicological Sciences* **2007,** *95* (1), 5-12.

8. Judson Richard, S.; Houck Keith, A.; Kavlock Robert, J.; Knudsen Thomas, B.; Martin Matthew, T.; Mortensen Holly, M.; Reif David, M.; Rotroff Daniel, M.; Shah, I.; Richard Ann, M.; Dix David, J., In Vitro Screening of Environmental Chemicals for Targeted Testing Prioritization: The ToxCast Project. *Environmental Health Perspectives* **2010,** *118* (4), 485-492.

9. Gadaleta, D.; Vuković, K.; Toma, C.; Lavado, G. J.; Karmaus, A. L.; Mansouri, K.; Kleinstreuer, N. C.; Benfenati, E.; Roncaglioni, A., SAR and QSAR modeling of a large collection of LD50 rat acute oral toxicity data. *Journal of Cheminformatics* **2019,** *11* (1), 58.

10. Abbott, B. D., Review of the expression of peroxisome proliferator-activated receptors alpha (PPARα), beta (PPARβ), and gamma (PPARγ) in rodent and human development. *Reproductive Toxicology* **2009,** *27* (3), 246-257.

11. Mansouri, K.; Grulke, C. M.; Judson, R. S.; Williams, A. J., OPERA models for predicting physicochemical properties and environmental fate endpoints. *Journal of Cheminformatics* **2018,** *10* (1), 10.
